# Astrocytic PERK Deficiency Drives Prefrontal Circuit Dysfunction and Depressive‐Like Behaviors

**DOI:** 10.1002/advs.202510780

**Published:** 2025-11-30

**Authors:** Kai Chen, Riya Gupta, Yosuke M. Morizawa, Yu Qin, Xingyu Du, Cynthia Pang, Osama Al‐Dalahmah, Maura B. Dupont, Guang Yang

**Affiliations:** ^1^ Department of Anesthesiology Columbia University Irving Medical Center New York NY 10032 USA; ^2^ Barnard College of Columbia University New York NY 10025 USA; ^3^ Department of Cell Biology and Neuroscience Rutgers, The State University of New Jersey Piscataway NJ 08854 USA; ^4^ Department of Pathology and Cell Biology Columbia University Irving Medical Center New York NY 10032 USA; ^5^ Department of Psychiatry Columbia University Irving Medical Center New York NY 10032 USA

**Keywords:** astrocyte, depression, gene therapy, Nrf2, PERK, prefrontal cortex, synaptic plasticity, TSP1

## Abstract

Major depressive disorder (MDD) is associated with dysfunction in prefrontal cortex (PFC) circuits, yet the glial mechanisms underlying these abnormalities remain unclear. Here, downregulation of the endoplasmic reticulum (ER) stress sensor PERK in PFC astrocytes is identified as a mechanistic contributor to depression‐related phenotypes. PERK expression is markedly reduced in PFC astrocytes from individuals with MDD and in two chronic‐stress mouse models. Astrocyte‐specific PERK deletion in stress‐naïve mice is sufficient to induce robust depressive‐like behaviors and widespread PFC circuit pathology, including dendritic spine loss, pyramidal neuron hypoactivity, and weakened functional connectivity. Mechanistically, PERK‐deficient astrocytes display reduced Nrf2 abundance, dysregulated ER and cytosolic Ca^2+^ dynamics, and decreased expression of the synaptogenic protein thrombospondin‐1 (TSP1). Restoring astrocytic TSP1 via a blood‐brain barrier‐penetrant adeno‐associated virus rescues PFC circuit function and reverses depressive‐like behaviors. These findings establish astrocytic PERK deficiency as a sufficient driver of synaptic and network dysfunction underlying depressive phenotypes and highlight astrocyte‐directed TSP1 augmentation as a potential therapeutic strategy for MDD.

## Graphical abstract

Chen et al. show that the endoplasmic reticulum (ER) stress sensor PERK is downregulated in prefrontal cortex (PFC) astrocytes in major depressive disorder and in chronic‐stress mouse models. In young mice, astrocyte‐specific PERK loss reduces the synaptogenic cue thrombospondin‐1 (TSP1), leading to synaptic and circuit deficits and depressive‐like behaviors. Restoring astrocytic TSP1 rescues PFC circuit function and reverses these depressive phenotypes.

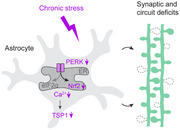

## 1 Introduction

Major depressive disorder (MDD) is a debilitating neuropsychiatric illness characterized by persistent low mood, anhedonia, and elevated suicide risk.^[^ ^1^, ^2^ ^]^ Clinical and preclinical studies have implicated dysfunction in neural circuits that support emotional and cognitive processing in the pathophysiology of MDD.^[^ ^3^, ^4^ ^]^ Among these circuits, the prefrontal cortex (PFC) is consistently affected,^[^ ^5^ ^]^ exhibiting reduced synapse density, aberrant neuronal activity, and disrupted functional connectivity.^[^ ^6^, ^7^, ^8^, ^9^ ^]^

Astrocytes, the most abundant glial cell type in the brain, are increasingly recognized as active modulators of mood‐related circuits.^[^ ^10^, ^11^ ^]^ By sensing neurotransmitter release, astrocytes engage intracellular Ca^2+^ signaling, which shapes gliotransmitter output, thereby tuning synaptic transmission and network dynamics.^[^ ^12^ ^]^ Disruption of astrocyte function induces depressive‐like behaviors in rodents.^[^ ^13^, ^14^, ^15^, ^16^, ^17^ ^]^ For example, developmental suppression of astrocytic Ca^2+^ signaling through overexpression of the plasma membrane Ca^2+^‐transporting ATPase 2 (PMCA2) produces persistent social deficits, depressive‐like behaviors, and synaptic abnormalities.^[^ ^18^ ^]^ Similarly, mice lacking the inositol 1,4,5‐trisphosphate receptor type 2 (IP3R2), a principal mediator of endoplasmic reticulum (ER) Ca^2+^ release in astrocytes, exhibit depressive‐like behaviors and disrupted resting‐state functional connectivity in PFC‐related networks, mirroring findings in individuals with MDD.^[^ ^14^, ^19^ ^]^ Consistent with these observations, postmortem studies report reduced expression of astrocyte markers (e.g., GFAP, S100β) in the PFC of individuals with MDD,^[^ ^20^ ^]^ and single‐nucleus RNA sequencing (RNA‐seq) studies have reported enrichment of stress‐response pathways, including the unfolded protein response (UPR), in MDD astrocytes.^[^ ^21^ ^]^

Protein kinase R‐like ER kinase (PERK) is an ER stress sensor and a key component of the UPR.^[^ ^22^ ^]^ Upon activation, PERK phosphorylates the α subunit of eukaryotic initiation factor 2 (eIF2α), transiently reducing global translation to limit the unfolded protein burden,^[^ ^23^ ^]^ and activates nuclear factor erythroid 2‐related factor 2 (Nrf2) to drive antioxidant gene programs and enhance stress resilience.^[^ ^24^, ^25^ ^]^ Astrocytes lacking PERK are more vulnerable to ER stress.^[^ ^26^ ^]^ Beyond proteostasis, PERK regulates intracellular Ca^2+^ dynamics across multiple cell types, including pancreatic β‐cells and cortical neurons, thereby influencing insulin secretion, neuronal excitability, and behavioral flexibility.^[^ ^27^, ^28^, ^29^, ^30^, ^31^ ^]^ Whether PERK similarly controls astrocytic Ca^2+^ signaling and contributes to depression‐related pathology remains unknown.

In this study, we show that PERK expression is markedly reduced in PFC astrocytes from individuals with MDD and in chronic‐stress mouse models. Astrocyte‐specific PERK deletion in otherwise unstressed mice is sufficient to induce robust depressive‐like behaviors and widespread PFC circuit abnormalities, including dendritic spine loss, pyramidal neuron hypoactivity, and weakened functional connectivity. At the molecular level, PERK deficiency lowers Nrf2 abundance, disrupts astrocytic ER and cytosolic Ca^2+^ signaling, and downregulates thrombospondin‐1 (TSP1), an astrocyte‐derived synaptogenic factor.^[^ ^32^ ^]^ Notably, systemic delivery of a blood‐brain barrier (BBB)‐penetrant adeno‐associated virus (AAV) to restore astrocytic TSP1 expression rescues PFC circuit function and reverses depressive‐like behaviors. Together, these findings identify astrocytic PERK downregulation as a mechanistic contributor to MDD‐related pathology and highlight TSP1 restoration as a promising astrocyte‐targeted therapeutic strategy.

## 2 Results

### 2.1 Astrocytic PERK is Downregulated in Individuals with MDD and in Chronic‐Stress Mouse Models

**Figure 1:**
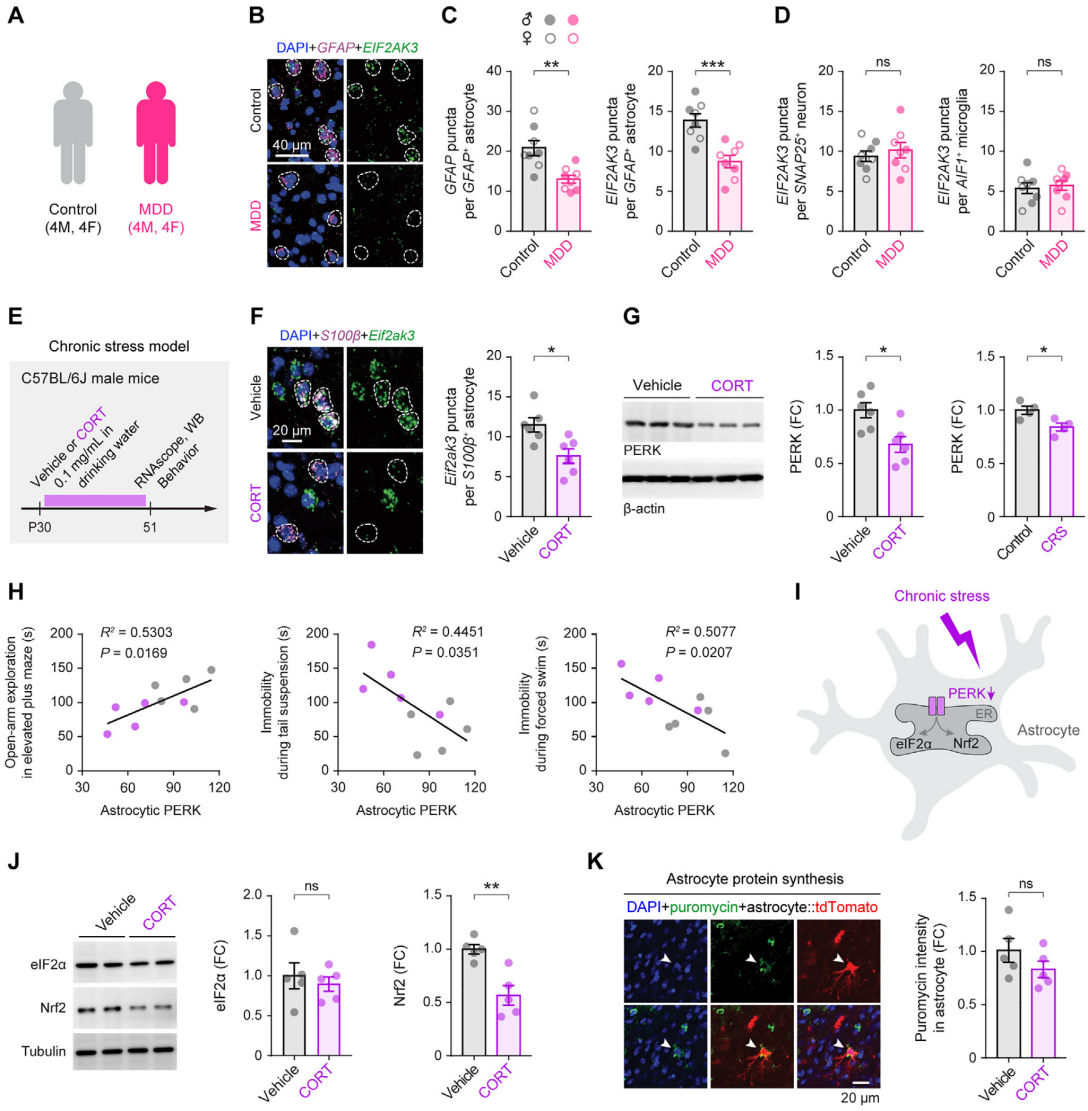
Astrocytic PERK signaling is reduced in the prefrontal cortex of individuals with MDD and in chronically stressed mice. **A**) RNAscope fluorescence in situ hybridization in postmortem anterior prefrontal cortex (BA10) from control subjects (*n* = 8; 4 male, 4 female; ages 54–95 years) and individuals with MDD (*n* = 8; 4 male, 4 female; ages 41–85 years). **B**) Representative RNAscope images showing *EIF2AK3* and *GFAP* transcripts with DAPI nuclear counterstaining. *GFAP* ^+^ astrocyte somata are outlined by dashed lines. **C**) Quantification of *GFAP* puncta per astrocyte (*t*_14_ = 3.622, *P* = 0.0028) and *EIF2AK3* puncta within *GFAP* ^+^ astrocytes (*t*_14_ = 4.515, *P* = 0.0005). **D**) *EIF2AK3* puncta in *SNAP25* ^+^ neurons (*t*_14_ = 0.6665, *P* = 0.5159) and *AIF1* ^+^ microglia (*t*_14_ = 0.3888, *P* = 0.7033). **E**) Experimental timeline for mouse chronic‐stress paradigms. **F**) Left, representative RNAscope images showing *Eif2ak3* and *S100β* in the medial prefrontal cortex (mPFC) of wild‐type mice treated with vehicle or corticosterone (CORT) (*n* = 6 mice/group). Right, quantification of *Eif2ak3* puncta in *S100β*⁺ astrocytes (*t*_10_ = 3.047, *P* = 0.0123). **G**) Immunoblot analysis of PERK protein in astrocytes isolated from mPFC after chronic CORT (*n* = 6 mice/group; *t*_10_ = 3.099, *P* = 0.0113) or chronic restraint stress (CRS) (*n* = 4 mice/group; *t*_6_ = 2.908, *P* = 0.0270). **H**) Pearson correlations between astrocytic PERK protein levels and measures of anxiety‐ and depressive‐like behaviors in mice. **I**) Schematic of canonical PERK signaling pathways. **J**) Immunoblot analysis of total eIF2α (*t*_8_ = 0.5598, *P* = 0.591) and Nrf2 (*t*_8_ = 4.259, *P* = 0.0028) in mPFC astrocytes (*n* = 5 mice/group). **K**) Immunofluorescent detection of puromycin incorporation (SUnSET assay) in mPFC astrocytes as a readout of de novo protein synthesis (*n* = 5 mice/group; *t*_8_ = 1.318, *P* = 0.2238). Summary data are mean ± SEM. *P < 0.05, **P < 0.01, ***P < 0.001; ns, not significant; two‐tailed unpaired *t* test. Each dot represents one biological replicate (human subject or mouse). FC, fold change.

To test whether astrocytic PERK signaling is altered in depression, we performed RNAscope fluorescence in situ hybridization (RNAscope FISH) on postmortem human anterior PFC (Brodmann area 10) samples from individuals with MDD and age‐matched controls (Table S1, Supporting Information). As previously reported,^[^ ^33^, ^34^ ^]^ *GFAP* transcripts were significantly reduced in MDD astrocytes (**Figure** **1**A–C). Critically, *EIF2AK3* (encoding PERK) transcripts were also significantly decreased specifically within GFAP^+^ astrocytes (Figure 1C), with no changes in *SNAP*25⁺ neurons or *AIF1*⁺ microglia (Figure 1D), indicating an astrocyte‐selective reduction of PERK expression in MDD. No sex‐dependent differences in astrocytic *EIF2AK3* were observed (Figure 1C,D).

We next asked whether chronic stress produces a similar signature in mice. Male C57BL/6J mice were subjected to two established stress paradigms: chronic corticosterone (CORT) exposure (Figure 1E)^[^ ^8^, ^35^ ^]^ and chronic restraint stress (CRS).^[^ ^36^ ^]^ As expected, CORT (0.1 mg/mL in drinking water from postnatal day 30–51) elicited robust anxiety‐ and depressive‐like behaviors across the open field, elevated plus maze, tail suspension, forced swim, and sucrose preference tests (Figure S1A,B, Supporting Information). In these mice, *Eif2ak3* transcripts were significantly reduced in prelimbic (PL) medial PFC (mPFC) astrocytes (Figure 1F), and immunoblotting of sorted mPFC astrocytes confirmed a corresponding decrease in PERK protein (Figure 1G; Figure S1C, Supporting Information). PERK abundance was inversely correlated with the severity of behavioral measures of anxiety‐ and depressive‐like phenotypes (Figure 1H). CRS yielded similar reductions in astrocytic PERK protein (Figure 1G).

PERK coordinates adaptive ER stress responses by phosphorylating eIF2α to transiently suppress global translation^[^ ^23^ ^]^ and by activating Nrf2 to induce antioxidant programs^[^ ^24^, ^25^ ^]^ (Figure 1I). In mPFC astrocytes from CORT‐treated mice, total eIF2α levels and de novo protein synthesis were unchanged (Figure 1J,K), whereas Nrf2 protein was significantly reduced (Figure 1J), consistent with the lower Nrf2 levels reported in the PFC of individuals with MDD.^[^ ^37^ ^]^ Together, human and rodent data converge on an astrocyte‐specific attenuation of PERK signaling in MDD and chronic stress, implicating astrocytic PERK loss as a mechanistic contributor to depression‐related pathology.

### 2.2 Astrocyte‐Specific PERK Deletion Induces Depressive‐Like Behaviors

**Figure 2:**
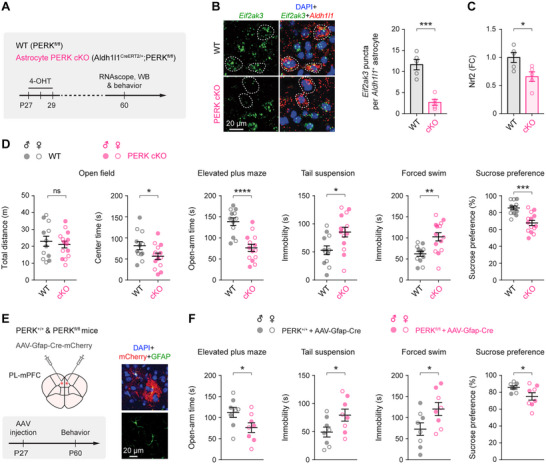
Astrocyte‐specific PERK deletion induces depressive‐like behaviors in naïve mice. **A**) Experimental timeline showing 4‐hydroxytamoxifen (4‐OHT) administration to induce astrocyte‐specific conditional PERK knockout (PERK cKO; Aldh1l1^CreERT2/+^;PERK^fl/fl^), followed by molecular and behavioral analyses. **B**) Left, representative RNAscope images of *Eif2ak3* in the mPFC. Right, quantification of *Eif2ak3* puncta per *Aldh1l1* ^+^ astrocyte (*n* = 5 mice/group; *t*_8_ = 6.323, *P* = 0.0002). **C**) Immunoblot analysis of Nrf2 protein in astrocytes isolated from the mPFC (*n* = 5 mice/group; *t*_8_ = 2.779, *P* = 0.0240). **D**) Behavioral performance of PERK^fl/fl^ (WT; *n* = 12 mice) and Aldh1l1^CreERT2/+^;PERK^fl/fl^ (PERK cKO; *n* = 14 mice) mice: open field (total distance traveled: *t*_24_ = 0.5507, *P* = 0.5869; center time: *t*_24_ = 2.091, *P* = 0.0473), elevated plus maze (open‐arm time, *t*_24_ = 5.07, *P* < 0.0001), tail suspension (immobility, *t*_24_ = 2.742, *P* = 0.0114), forced swim (immobility, *t*_24_ = 3.402, *P* = 0.0023), and sucrose preference (*t*_24_ = 4.563, *P* = 0.0001). **E**) Left, schematic of prelimbic mPFC (PL‐mPFC)‐targeted astrocytic PERK deletion via bilateral AAV‐Gfap‐Cre injection, followed by behavioral testing. Right, representative confocal images showing robust mCherry expression in GFAP^+^ astrocytes within the mPFC. **F**) Behavioral outcomes after PL‐mPFC‐targeted astrocytic PERK deletion (*n* = 8 mice/group): elevated plus maze (*t*_14_ = 2.166, *P* = 0.0481), tail suspension (*t*_14_ = 2.17, *P* = 0.0477), forced swim (*t*_14_ = 2.203, *P* = 0.0449), and sucrose preference (*t*_14_ = 2.228, *P* = 0.0428). Summary data are mean ± SEM. ns, not significant; *P < 0.05, **P < 0.01, ***P < 0.001, ****P < 0.0001; two‐tailed unpaired *t* test. Each dot represents one mouse.

To test causality, we generated astrocyte‐specific conditional PERK knockout (PERK cKO) mice (Aldh1l1^CreERT2/+^;PERK^fl/fl^) by crossing Aldh1l1‐Cre/ER^T2^ mice^[^ ^38^ ^]^ with PERK^fl/fl^ mice. Cre recombination was induced with three consecutive daily injections of 4‐hydroxytamoxifen (4‐OHT; **Figure** **2**A). One month later, RNAscope confirmed a robust reduction of *Eif2ak3* transcripts in cortical astrocytes (Figure 2B), accompanied by decreased Nrf2 protein levels (Figure 2C), mirroring changes observed in chronically stressed wild‐type (WT) mice (Figure 1J).

Locomotor activity was unaffected in PERK cKO mice, as indicated by total distance traveled in the open field (Figure 2D). However, multiple anxiety‐ and depressive‐like phenotypes emerged, including reduced center exploration in the open field, decreased time spent in the open arms of the elevated plus maze, increased immobility in the tail suspension and forced swim tests, and diminished sucrose preference (Figure 2D). Auditory‐cued fear conditioning remained intact (Figure S2A,B, Supporting Information), indicating preserved associative memory.

Consistent with clinical observations,^[^ ^39^ ^]^ PERK cKO mice showed a selective reduction of the astrocytic calcium‐binding protein S100β in the mPFC (Figure S2C–E, Supporting Information), with no detectable changes in the somatosensory or visual cortex. To establish regional sufficiency, we bilaterally infused AAV‐Gfap‐Cre into the PL‐mPFC of PERK^fl/fl^ mice (Figure 2E). Astrocyte‐restricted Cre expression was validated using Ai14 reporter mice (Figure S2F, Supporting Information), and PERK loss in mPFC astrocytes was confirmed by immunoblotting (Figure S2G, Supporting Information). One month after injection, mice with mPFC‐restricted astrocytic PERK deletion exhibited robust anxiety‐ and depressive‐like phenotypes across assays (Figure 2F), closely resembling the global astrocytic PERK cKO profile. These data demonstrate that loss of PERK specifically in mPFC astrocytes is sufficient to drive depressive‐like behaviors in the absence of exogenous stressors.

### 2.3 PERK Deficiency Impairs Ca^2+^ Signaling in Astrocytes

**Figure 3:**
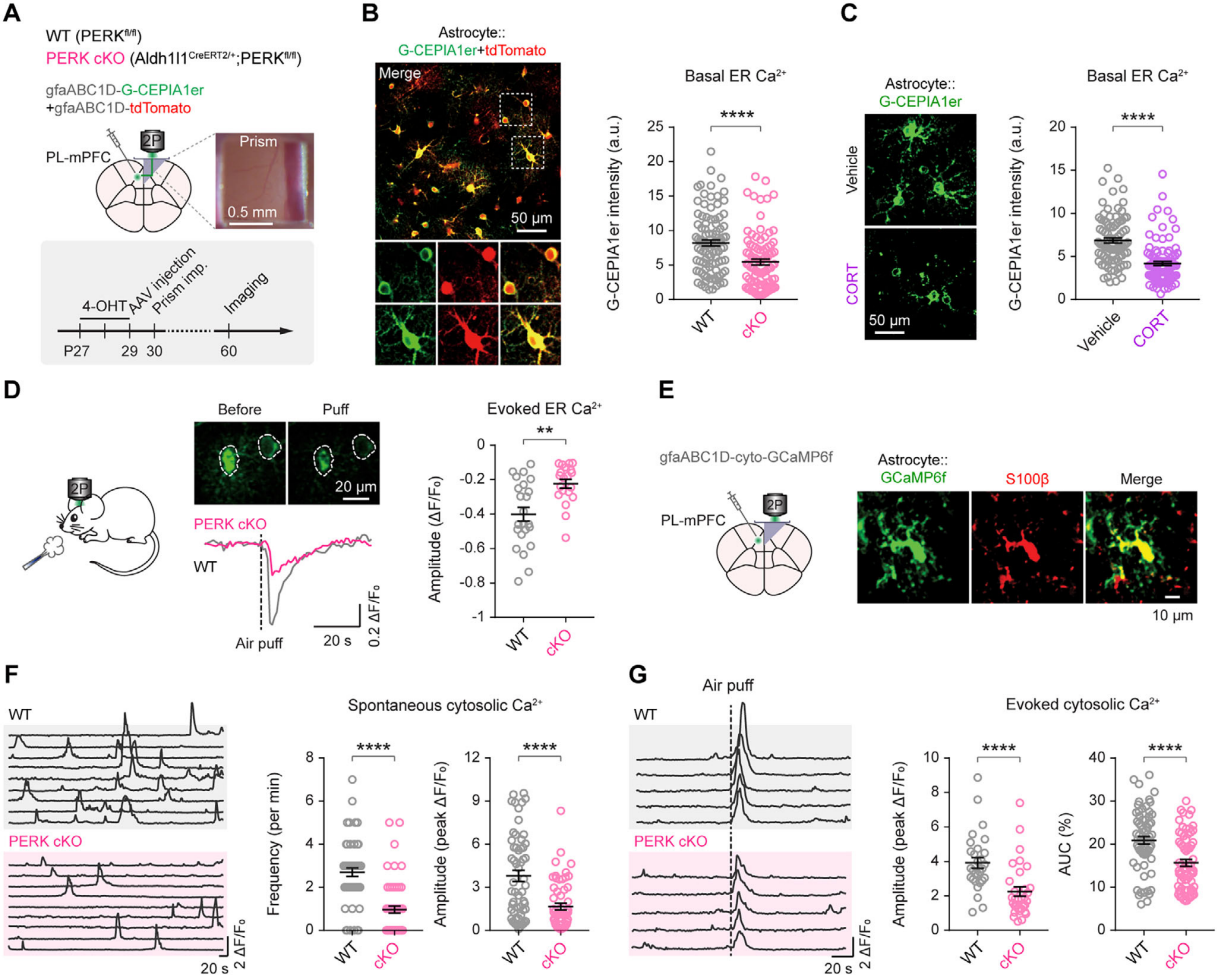
Astrocytic PERK deletion diminishes ER and cytosolic Ca^2+^ signaling in vivo. **A**) Experimental timeline for in vivo two‐photon imaging of ER Ca^2+^ dynamics in mPFC astrocytes following astrocyte‐specific PERK cKO. **B**) Left, representative two‐photon images from awake mice showing PERK‐deficient astrocytes expressing G‐CEPIA1er (ER Ca^2+^ sensor) and tdTomato (cytosolic marker); enlarged views of boxed regions are shown below. Right, quantification of baseline ER Ca^2+^ levels in astrocytes with and without PERK cKO (*n* = 95, 93 cells from 5 mice/group; *P* < 0.0001). **C**) ER Ca^2+^ levels were significantly reduced in astrocytes from mice exposed to chronic CORT, as measured by G‐CEPIA1er fluorescence (*n* = 89, 97 cells from 5 mice/group; *P* < 0.0001). **D**) Left, representative images and traces of air‐puff‐evoked ER Ca^2+^ responses in astrocytes. Right, peak ER Ca^2+^ response amplitude (*n* = 23, 20 cells from 5 mice/group; *P* = 0.0016). **E**) Experimental design for in vivo two‐photon imaging of cytosolic Ca^2+^ dynamics in mPFC astrocytes after astrocytic PERK deletion. **F**) Left, representative traces of spontaneous somatic Ca^2+^ transients. Right, frequency and amplitude of spontaneous Ca^2+^ events at rest (*n* = 57, 57 cells from 5 mice/group; frequency, *P* < 0.0001; amplitude, *P* < 0.0001). **G)** Left, representative traces of somatic Ca^2+^ responses to air‐puff stimulation (5 mice/group). Right, peak amplitude (*n* = 29, 32 cells; *P* < 0.0001) and area under the curve (AUC; *n* = 65, 67 cells; *P* < 0.0001) for evoked responses. Summary data are mean ± SEM. **P < 0.01, ****P < 0.0001; Mann‐Whitney U test. Each dot represents one cell. a.u., arbitrary units.

Dysregulated astrocytic Ca^2+^ signaling has been implicated in depressive‐like behaviors,^[^ ^18^, ^19^, ^40^ ^]^ and PERK contributes to the regulation of ER Ca^2+^ homeostasis.^[^ ^28^ ^]^ To test whether PERK loss disrupts ER Ca^2+^ dynamics in vivo, we expressed the ER‐targeted Ca^2+^ sensor G‐CEPIA1er^[^ ^41^ ^]^ together with cytosolic tdTomato under the astrocyte‐specific gfaABC1D promoter in the mPFC. We performed two‐photon imaging in awake mice through a chronically implanted microprism contralateral to the injection site (**Figure** **3**A; Figure S3A, Supporting Information). As expected, G‐CEPIA1er localized to tdTomato^+^ astrocyte somata (Figure 3B), and sensor responsiveness was validated (Figure S3B, Supporting Information).

PERK‐deficient astrocytes exhibited significantly lower baseline G‐CEPIA1er fluorescence than controls (Figure 3B), indicating reduced ER Ca^2+^ content – an effect that was not attributable to differences in sensor expression (Figure S3C, Supporting Information). WT mice exposed to chronic CORT showed a similar reduction in ER Ca^2+^ levels (Figure 3C), suggesting that impaired ER Ca^2+^ signaling is a common feature of depression‐related conditions. To assess stimulus‐evoked ER Ca^2+^ release, we used an air‐puff startle paradigm that activates α_1_‐adrenergic receptors and engages IP_3_‐dependent ER Ca^2+^ mobilization.^[^ ^42^ ^]^ In WT controls, air puffs evoked transient decreases in ER Ca^2+^ signals (Figure 3D), whereas PERK‐deficient astrocytes displayed markedly attenuated responses, indicating impaired Ca^2+^ mobilization.

Because the ER is the principal intracellular Ca^2+^ reservoir, we next asked whether reduced ER Ca^2+^ availability might alter cytosolic Ca^2+^ dynamics. Expression of the cytosolic indicator GCaMP6f in mPFC astrocytes (Figure 3E) revealed significantly lower frequencies and amplitudes of spontaneous cytosolic Ca^2+^ transients at rest (Figure 3F), as well as attenuated air‐puff‐evoked responses (Figure 3G) in PERK‐deficient astrocytes compared with PERK^fl/fl^ controls. Together, these findings demonstrate that PERK is essential for sustaining astrocytic Ca^2+^ signaling in both the ER and cytosolic compartments in vivo.

### 2.4 Astrocytic PERK Deletion Leads to Synapse Loss

**Figure 4:**
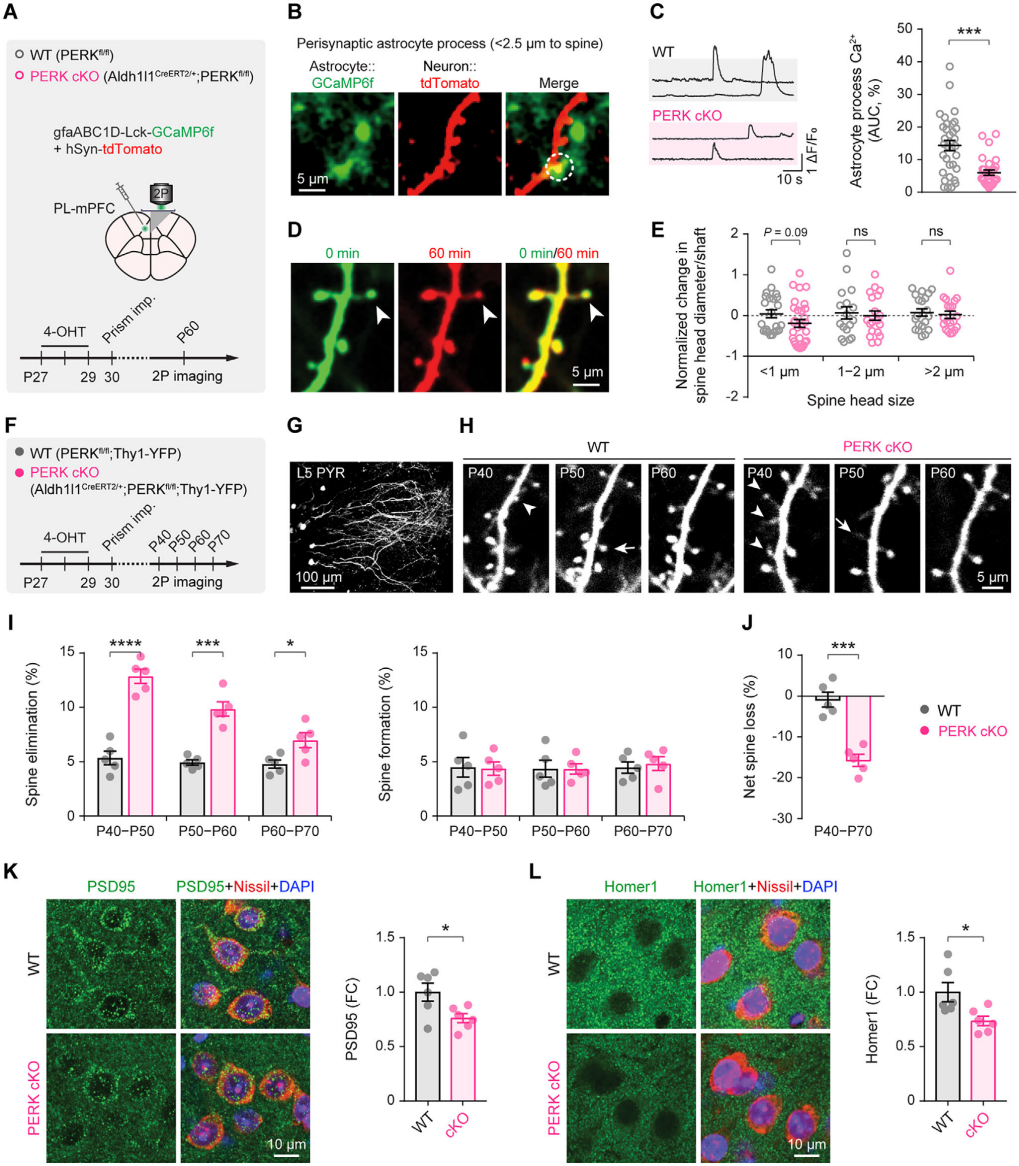
Astrocytic PERK deletion reduces perisynaptic astrocyte Ca^2+^ activity and promotes synapse loss. **A**) Experimental design for in vivo two‐photon Ca^2+^ imaging of perisynaptic astrocyte processes surrounding dendritic spines in the mPFC. **B**) Representative two‐photon images showing astrocytes expressing Lck‐GCaMP6f and neuronal dendrites labeled with tdTomato. **C**) Left, representative Ca^2+^ traces from perisynaptic astrocytic processes. Right, quantification of perisynaptic astrocyte Ca^2+^ activity (2‐min AUC) within 2.5 µm of dendritic spine heads (*n* = 33, 30 spines from 5 mice/group; *P* = 0.0001). **D**) Time‐lapse images of the same dendritic segments acquired over a 60‐min imaging session. **E**) Changes in spine head size during the imaging session (<1 µm, *t*_51_ = 1.68, *P* = 0.0991; 1–2 µm, *t*_34_ = 0.3434, *P* = 0.7334; >2 µm, *t*_38_ = 0.401, *P* = 0.6907). **F**) Experimental design and timeline for longitudinal in vivo two‐photon imaging of dendritic spines in Thy1‐YFP mice with or without astrocytic PERK cKO. **G**) Two‐photon image showing apical dendrites of layer 5 (L5) pyramidal neurons (PYRs) in the mPFC. **H**) Representative images of apical dendrites over a 10‐day interval; arrows indicate newly formed spines; arrowheads indicate eliminated spines. **I**) Time course of dendritic spine dynamics (*n* = 5 mice/group): spine elimination (P40–P50, *t*_8_ = 8.245, *P* < 0.0001; P50–P60, *t*_8_ = 6.922, *P* = 0.0001; P60–P70, *t*_8_ = 2.824, *P* = 0.0224) and spine formation (P40–P50, *t*_8_ = 0.1079, *P* = 0.9168; P50–P60, *t*_8_ = 0.02677, *P* = 0.9793; P60–P70, *t*_8_ = 0.4305, *P* = 0.6782). **J**) Net dendritic spine loss across P40–P70 (*t*_8_ = 6.352, *P* = 0.0002). **K**) Immunostaining and quantification of PSD95 puncta in the mPFC (*n* = 6 mice/group; *t*_10_ = 2.605, *P* = 0.0263). **L**) Immunostaining and quantification of Homer1 puncta in the mPFC (*n* = 6 mice/group; *t*_10_ = 2.697, *P* = 0.0224). Summary data are mean ± SEM. *P < 0.05, ***P < 0.001, ****P < 0.0001; Mann‐Whitney U test (**C**) or two‐tailed unpaired *t* test (**E**, **I**–**L**). Each dot represents an individual spine (**C**, **E**) or an individual animal (**I–L**).

Astrocytic processes, which constitute ∼70–80% of the astrocytic plasma membrane, form extensive contacts with neuronal synapses,^[^ ^43^, ^44^ ^]^ and perisynaptic Ca^2+^ signaling is crucial for bidirectional neuron‐astrocyte communication.^[^ ^45^, ^46^ ^]^ To determine whether PERK deficiency disrupts local perisynaptic Ca^2+^ dynamics, we sparsely labeled astrocytes with membrane‐tethered Lck‐GCaMP6f and cortical neurons with tdTomato in PERK cKO mice (**Figure** **4**A). This approach enabled simultaneous monitoring of astrocytic Ca^2+^ microdomains within 2.5 µm of dendritic spine heads while simultaneously tracking spine morphology. Consistent with somatic Ca^2+^ deficits (Figure 3E–G), PERK‐deficient astrocytes exhibited significantly reduced perisynaptic Ca^2+^ activity compared with controls (Figure 4B,C). Spine head size remained stable over a 60‐min imaging session, although thin spines (head diameter < 1 µm) in PERK cKO mice showed a nonsignificant trend toward smaller head size (Figure 4D,E).

To assess longer‐term synaptic structural consequences, we performed longitudinal two‐photon imaging in PERK cKO × Thy1‐YFP‐H mice, which express YFP in layer 5 pyramidal neurons (Figure 4F–H). Over 10 days, PERK cKO mice exhibited a significant increase in dendritic spine elimination without a change in spine formation (Figure 4I). This elevated elimination was most pronounced early after PERK deletion (P40–P50) and diminished at later stages (P60–P70), resulting in an approximately 15% net reduction in total spine number in the mPFC between P40 and P70 (Figure 4J). Immunostaining corroborated these findings, revealing reduced PSD95 and Homer1 in the PERK cKO mPFC relative to controls (Figure 4K,L).

Collectively, these results are consistent with prior reports of dendritic retraction and synaptic loss in the PFC following chronic stress,^[^ ^47^, ^48^ ^]^ and support a model in which astrocytic PERK loss impairs perisynaptic Ca^2+^ signaling, thereby destabilizing synapses and promoting their elimination.

### 2.5 Astrocytic PERK Deletion Results in mPFC Circuit Dysfunction

**Figure 5:**
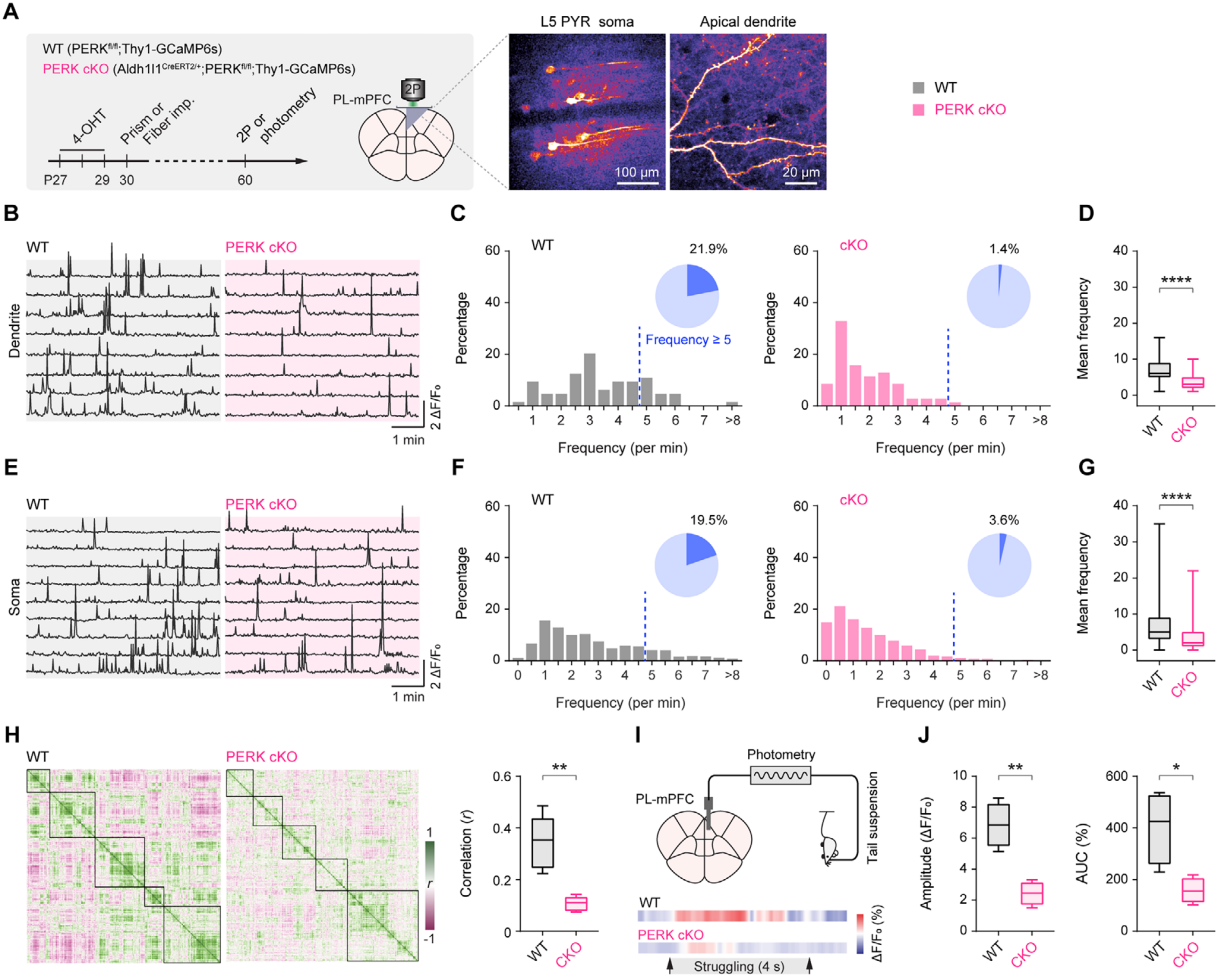
Astrocytic PERK deletion reduces pyramidal neuron activity and impairs functional connectivity in the mPFC. **A**) Left, experimental timeline for in vivo two‐photon Ca^2+^ imaging of pyramidal neurons (PYRs) in the mPFC of awake PERK^fl/fl^ (WT) and Aldh1l1^CreERT2/+^;PERK^fl/fl^ (PERK cKO) mice. Right, representative two‐photon images of L5 PYRs expressing GCaMP6s. **B**) Representative 5‐min dendritic Ca^2+^ traces from L5 PYRs. **C**) Frequency distribution of dendritic Ca^2+^ transients in PYRs (*n* = 64, 70 dendrites from 5 mice/group). **D**) Mean dendritic Ca^2+^ transient frequency is reduced in PERK cKO mice (*U* = 900, *P* < 0.0001). **E**–**G**) Analogous analyses of somatic Ca^2+^ activity in PYRs (*n* = 1061, 987 neurons from 5 mice/group; *U* = 291 825, *P* < 0.0001). **H**) Left, representative Pearson correlation matrices of somatic Ca^2+^ activity across simultaneously imaged, active L5 PYRs (*n* = 5 mice/group). Right, mean pairwise correlation coefficients are reduced in PERK cKO mice (*t*_8_ = 4.959, *P* = 0.0011). **I**) Schematic of fiber photometry for monitoring population Ca^2+^ signals from mPFC PYRs during the tail suspension test (*n* = 4 mice/group). **J**) Population Ca^2+^ responses at struggle onset: peak amplitude (*t*_6_ = 5.488, *P* = 0.0015) and AUC (4‐s window; *t*_6_ = 3.302, *P* = 0.0164) are reduced in PERK cKO mice. Summary data are mean ± SEM. *P < 0.05, **P < 0.01, ****P < 0.0001; Mann‐Whitney U test (**D**, **G**) or two‐tailed unpaired *t* test (**H**, **J**). Box plot bounds and center, quartiles and median; whiskers, min and max.

To quantify circuit‐level consequences, we performed two‐photon Ca^2+^ imaging of individual layer 5 pyramidal neurons in PERK cKO × Thy1.2‐GCaMP6s mice, which express GCaMP6s in excitatory neurons (**Figure** **5**A; Video S1, Supporting Information). Reduced mPFC neuronal activity and connectivity have been consistently associated with depressive‐like behaviors.^[^ ^6^, ^7^, ^8^, ^9^ ^]^ Compared with controls, PERK cKO mice exhibited significantly lower frequencies of Ca^2+^ transients in dendritic compartments (Figure 5B–D) and in somata (Figure 5E–G). The fraction of dendrites and somata showing sustained or bursting activity (≥5 Ca^2+^ transients per minute) was also reduced in PERK cKO mice.

Functional connectivity, computed from pairwise correlations of Ca^2+^ activity across simultaneously imaged neurons, was markedly diminished in PERK cKO mice (Figure 5H), indicating impaired local network coordination. To assess behaviorally relevant recruitment of mPFC circuits, we recorded bulk Ca^2+^ signals using fiber photometry during the tail suspension test (Figure 5I). In controls, mPFC activity increased during active struggle bouts,^[^ ^49^ ^]^ whereas PERK cKO mice showed significantly attenuated activation during these epochs (Figure 5J). Together, these findings indicate that astrocytic PERK deletion lowers neuronal activity, weakens functional connectivity, and attenuates behaviorally evoked mPFC responses.

### 2.6 PERK‐Deficient Astrocytes Downregulate TSP1, an Astrocyte‐Derived Synaptogenic Cue

**Figure 6:**
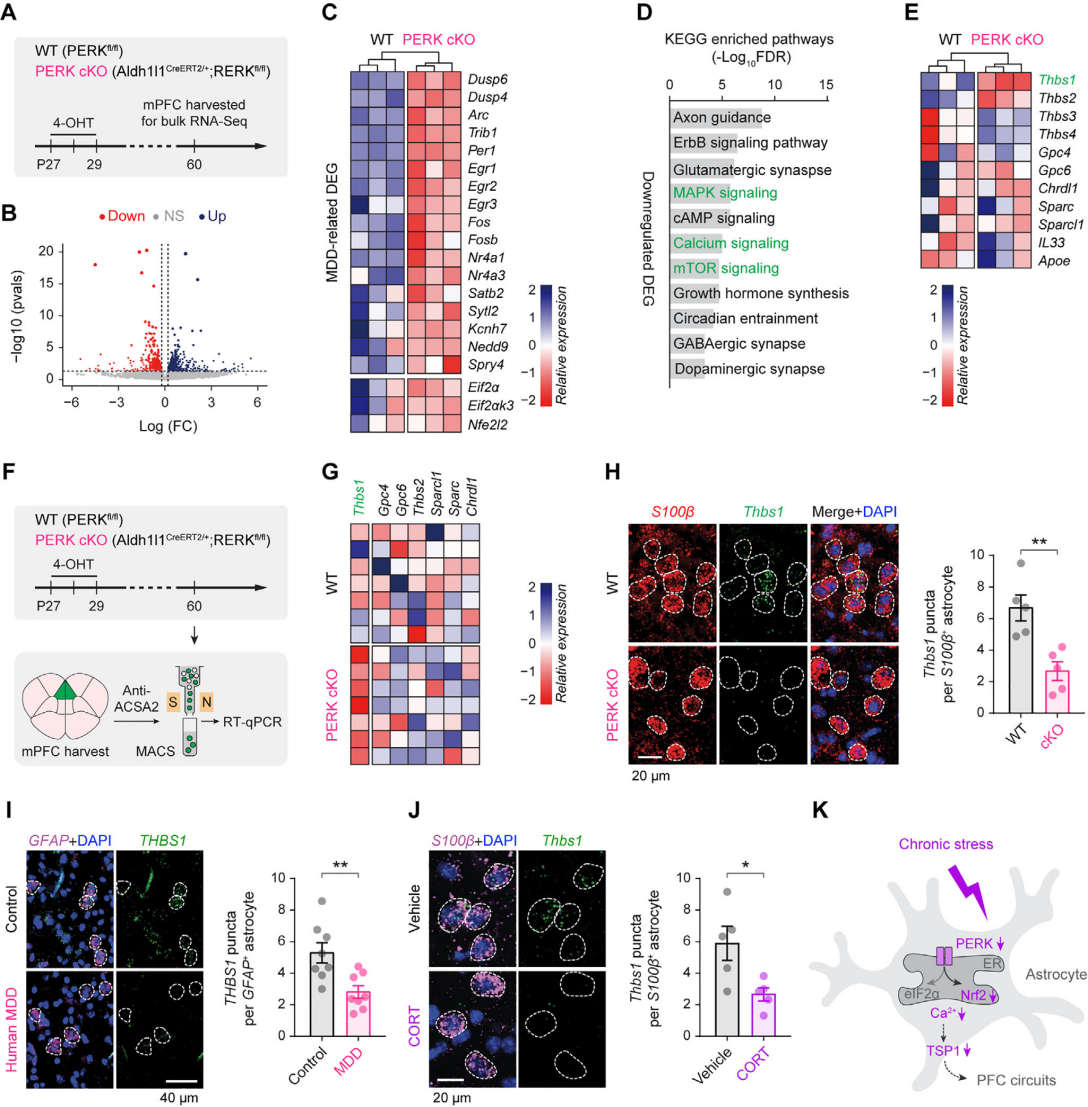
PERK‐deficient astrocytes downregulate the synaptogenic protein TSP1. **A**) Experimental timeline for bulk RNA‐seq of mPFC tissue from PERK^fl/fl^ (WT) and Aldh1l1^CreERT2/+^;PERK^fl/fl^ (astrocyte‐specific PERK cKO) mice. **B**) Volcano plot of differentially expressed genes (DEGs) comparing PERK cKO and WT mice (*n* = 3 mice/group). (**C**) Relative expression of MDD‐associated DEGs in the mPFC of PERK cKO and WT mice. **D**) KEGG pathway enrichment analysis for downregulated DEGs, highlighting MAPK, Ca^2+^, and mTOR signaling pathways, which are frequently altered in MDD. **E**) *Thbs1* (encoding TSP1) shows a trend toward lower expression in the PERK cKO mPFC (*P* = 0.0715). **F**) Experimental timeline for MACS‐based isolation of mPFC astrocytes followed by RT‐qPCR. **G**) RT‐qPCR confirms reduced *Thbs1* mRNA in PERK‐deficient astrocytes (*n* = 7 mice/group). **H**) Left, representative RNAscope images showing *Thbs1* puncta in *S100β*⁺ astrocytes of the mouse mPFC (*n* = 5 mice/group). Right, quantification (*t*_8_ = 3.919, *P* = 0.0044). **I**) Left, RNAscope images of *THBS1* in *GFAP*⁺ astrocytes from human anterior PFC (*n* = 8 subjects/group). Right, quantification (*t*_14_ = 3.305, *P* = 0.0052). **J**) Left, RNAscope images of *Thbs1* in *S100β* ^+^ astrocytes from the mPFC of mice exposed to chronic CORT (*n* = 5 mice/group). Right, quantification (*t*_8_ = 2.785, *P* = 0.0237). **K**) Working model: astrocytic PERK deficiency lowers Nrf2 expression and impairs Ca^2+^ signaling, leading to TSP1 downregulation and resulting in PFC circuit dysfunction. Summary data are mean ± SEM. *P < 0.05, **P < 0.01; two‐tailed unpaired *t* test. Each dot represents one biological replicate (mouse or human subject).

To identify molecular mechanisms linking astrocytic PERK loss to mPFC dysfunction, we performed bulk RNA‐seq on mPFC tissue from mice with astrocyte‐specific PERK deletion (**Figure** **6**A). PERK loss resulted in broad transcriptomic remodeling, with ∼850 genes upregulated and ∼1100 downregulated (Figure 6B). Among the top 50 downregulated genes were several previously implicated in MDD and stress models,^[^ ^50^ ^]^ including *Dusp6*, *Arc*, *Trib1*, *Egr2*, *Fos, and Nfe2l2* (encoding Nrf2) (Figure 6C), indicating that astrocytic PERK loss recapitulates depression‐associated molecular signatures. Pathway analysis revealed significant downregulation of synaptic function and the MAPK, Ca^2+^, and mTOR signaling pathways (Figure 6D), which are frequently perturbed in MDD.^[^ ^21^, ^50^ ^]^

Among astrocyte‐enriched synaptogenic factors, *Thbs1* (encoding TSP1, an established astrocyte‐secreted protein that promotes excitatory synaptogenesis^[^ ^32^ ^]^) showed a strong trend toward lower expression in PERK cKO mPFC tissue (*P* = 0.0715; Figure 6E), although this did not reach statistical significance. Given that TSP1 expression is regulated by astrocytic Ca^2+^ signaling^[^ ^51^ ^]^ and PERK deficiency disrupts Ca^2+^ dynamics (Figures 3 and 4), TSP1 emerged as a compelling mechanistic link between PERK loss and synaptic deficits.

To establish cell‐type specificity, we isolated mPFC astrocytes by magnetic‐activated cell sorting (MACS; Figure 6F). RT‐qPCR confirmed a significant decrease in *Thbs1* mRNA in PERK‐deficient astrocytes compared with controls (Figure 6G), and RNAscope further corroborated this reduction at single‐cell resolution (Figure 6H). Importantly, *Thbs1/THBS1* downregulation was also observed in astrocytes from human anterior PFC in MDD (Figure 6I) and in mPFC astrocytes from CORT‐treated mice (Figure 6J), underscoring translational relevance across species and models. Collectively, these findings identify TSP1 downregulation as a key molecular consequence of astrocytic PERK deficiency, providing a mechanistic link from impaired astrocytic Ca^2+^ signaling to synaptic and circuit dysfunction (Figure 6K).

### 2.7 Restoring TSP1 Expression Rescues mPFC Function and Depressive‐Like Behaviors

**Figure 7:**
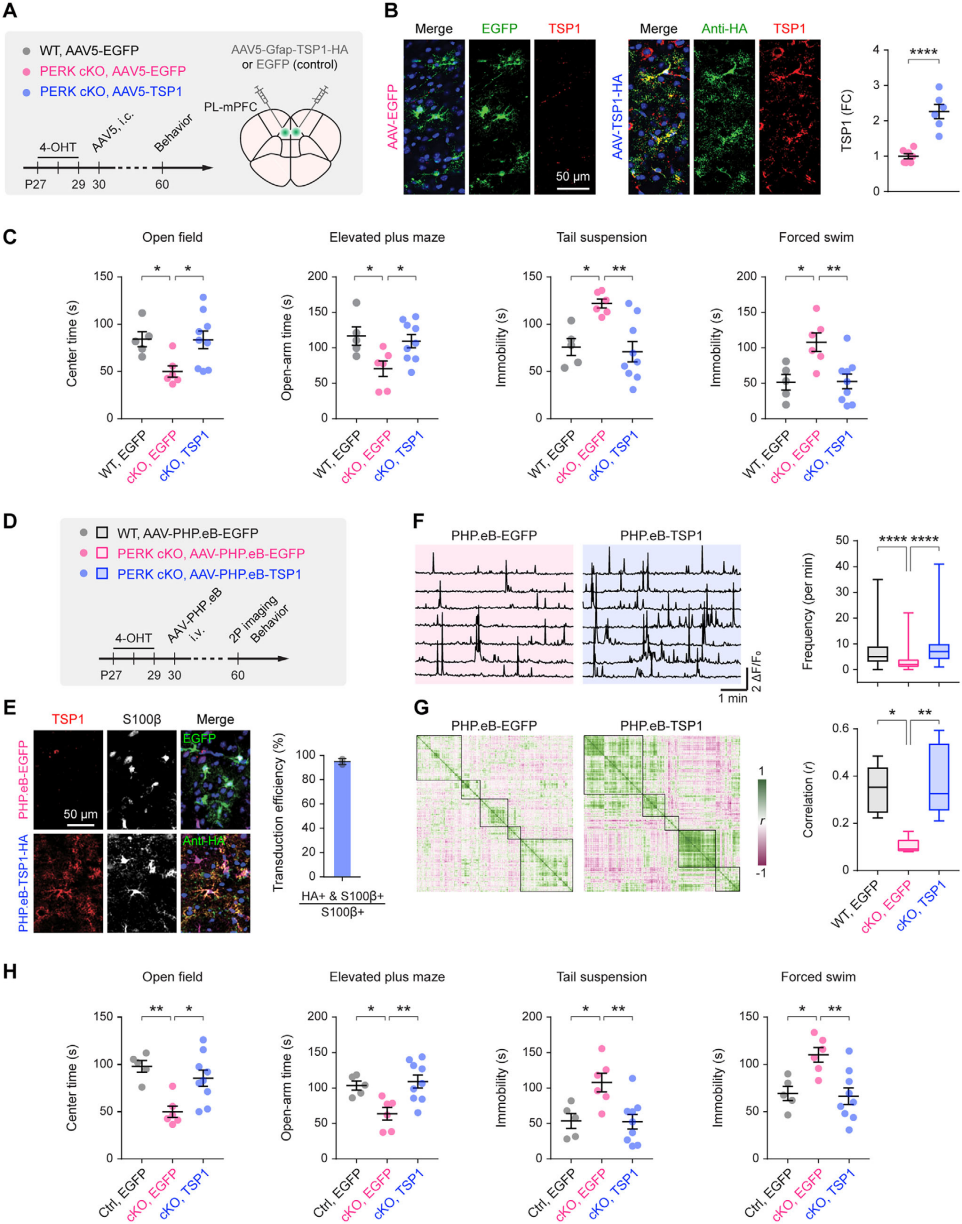
Astrocyte‐targeted TSP1 gene delivery restores mPFC circuit function and alleviates depressive‐like behaviors in PERK cKO mice. **A**) Experimental timeline and design for bilateral PL‐mPFC injections of AAV5‐Gfap‐TSP1‐HA or AAV5‐Gfap‐EGFP (control) in Aldh1l1^CreERT2/+^;PERK^fl/fl^ mice, followed by behavioral testing. **B**) Left, representative confocal images showing TSP1 immunoreactivity in the mPFC (*n* = 7, 6 mice). Right, quantification of astrocytic TSP1 expression in AAV5‐Gfap‐TSP1‐treated mice versus EGFP controls (*t*_11_ = 6.296, *P* < 0.0001). **C**) Behavioral outcomes (*n* = 5, 6, 9 mice): open field (*P* = 0.0494, *P* = 0.0240), elevated plus maze (*P* = 0.0291, *P* = 0.0333), tail suspension (*P* = 0.0133, *P* = 0.0022), and forced swim (*P* = 0.0136, *P* = 0.0055). **D**) Schematic of systemic delivery using the AAV‐PHP.eB vector to express TSP1 or EGFP (control) under the Gfap promoter. **E**) Left, confocal images of the mPFC showing astrocyte‐specific expression of HA‐tagged TSP1 colocalized with S100β. Right, percentage of S100β⁺ astrocytes expressing TSP1‐HA. **F**) Left, representative traces of somatic Ca^2+^ transients in L5 pyramidal neurons. Right, Ca^2+^ transient frequency across groups (*n* = 1061, 501, 529 neurons from 5 mice/group; *P* < 0.0001, *P* < 0.0001), indicating restoration of neuronal activity in TSP1‐treated PERK cKO mice. **G**) Left, correlation matrices of pyramidal neuron somatic Ca^2+^ activity. Right, mean pairwise correlation coefficients across groups (*P* = 0.0133; *P* = 0.0048), demonstrating enhanced functional connectivity following TSP1 rescue. **H**) Behavioral outcomes (*n* = 5, 6, 9 mice): open field (*P* = 0.0036, *P* = 0.0131), elevated plus maze (*P* = 0.0379, *P* = 0.0057), tail suspension (*P* = 0.0238, *P* = 0.0078), and forced swim (*P* = 0.0225, *P* = 0.0050). Summary data are mean ± SEM. *P < 0.05, **P < 0.01, ****P < 0.0001; two‐tailed unpaired *t* test (**B**) or one‐way ANOVA with Bonferroni's multiple‐comparisons test (**C**, **F**–**H**). Each dot represents one mouse. Box plot bounds and center, quartiles and median; whiskers, min and max.

To test whether TSP1 restoration can reverse phenotypes caused by astrocytic PERK loss, we used an astrocyte‐specific AAV5 vector (AAV5‐Gfap‐TSP1); control mice received AAV5‐Gfap‐EGFP. PERK cKO mice received bilateral mPFC injections (**Figure** **7**A). One month later, immunofluorescence revealed efficient astrocyte transduction (∼93%; Figure S4, Supporting Information) and approximately twofold higher mPFC TSP1 immunoreactivity in AAV5‐Gfap‐TSP1‐treated mice compared with EGFP controls (Figure 7B). Behaviorally, TSP1 overexpression significantly reduced anxiety‐ and depressive‐like phenotypes, as evidenced by increased center exploration in the open field, greater time spent in the open arms of the elevated plus maze, and decreased immobility in the tail suspension and forced swim tests (Figure 7C).

To enhance translational relevance, we next employed AAV‐PHP.eB,^[^ ^52^ ^]^ which enables broad CNS transduction via noninvasive intravenous delivery (Figure 7D). One month after a single retro‐orbital injection of AAV‐PHP.eB‐Gfap‐TSP1, robust TSP1 expression was detected in ≈95% of S100β⁺ astrocytes within the mPFC (Figure 7E), without evidence of BBB disruption (Figure S5, Supporting Information), consistent with prior reports.^[^ ^53^ ^]^ Systemic TSP1 restoration improved both circuit and behavioral outcomes: two‐photon imaging revealed significantly increased pyramidal neuron Ca^2+^ activity and strengthened functional connectivity in AAV‐PHP.eB‐Gfap‐TSP1‐treated PERK cKO mice compared with EGFP controls (Figure 7F,G), in parallel with increased synaptic marker expression (Figure S6A, Supporting Information). These circuit‐level improvements were accompanied by a robust rescue of depressive‐like behaviors (Figure 7H), with no changes in locomotion or mechanical sensitivity (Figure S6B,C, Supporting Information). Thus, restoring astrocytic TSP1, either locally or systemically, normalizes mPFC circuit function and reverses depressive‐like states in PERK cKO mice.

### 2.8 Systemic TSP1 Delivery via AAV‐PHP.eB Alleviates Depressive‐Like Behaviors in Chronic‐Stress Models

**Figure 8:**
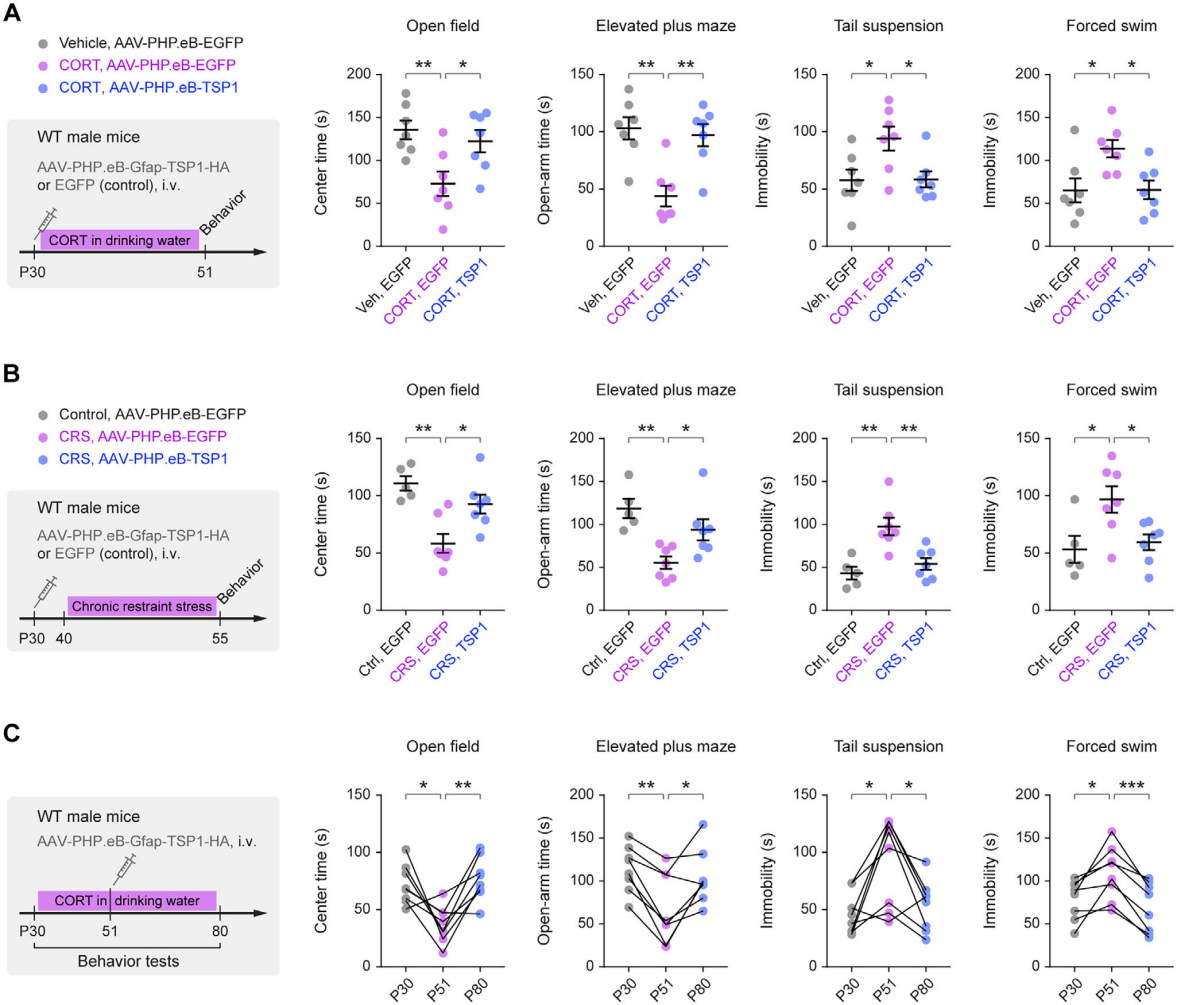
Astrocyte‐targeted TSP1 gene delivery prevents and reverses depressive‐like behaviors in chronic‐stress models. **A**) Left, experimental timeline for systemic AAV‐PHP.eB‐Gfap‐TSP1 delivery initiated at the onset of chronic CORT exposure (*n* = 7 mice/group). Right, behavioral performance showing that astrocytic TSP1 overexpression prevents the emergence of CORT‐induced depressive‐like phenotypes: open field (*P* = 0.008, 0.0392), elevated plus maze (*P* = 0.001, 0.0028), tail suspension (*P* = 0.0312, 0.0353), and forced swim (*P* = 0.0257, 0.0276). **B**) Left, experimental timeline for systemic TSP1 delivery prior to chronic restraint stress (CRS) (*n* = 5, 7, 7 mice). Right, behavioral performance showing prevention of CRS‐induced depressive‐like behaviors: open field (*P* = 0.0012, 0.0174), elevated plus maze (*P* = 0.0024, 0.0429), tail suspension (*P* = 0.0015, 0.0044), and forced swim (*P* = 0.0297, 0.0434). **C**) Left, experimental timeline testing therapeutic efficacy after the establishment of depressive‐like behaviors in mice previously exposed to chronic CORT (*n* = 8 mice). Right, behavioral performance demonstrating reversal of established phenotypes following systemic TSP1 delivery: open field (*P* = 0.0308, 0.0061), elevated plus maze (*P* = 0.0023, 0.0461), tail suspension (*P* = 0.0381, 0.0490), and forced swim test (*P* = 0.0401, 0.0005). Summary data are mean ± SEM. *P < 0.05, **P < 0.01, ***P < 0.001; one‐way ANOVA (**A**, **B**) or RM one‐way ANOVA (**C**) followed by Bonferroni's multiple‐comparisons test. Each dot represents one mouse.

To evaluate therapeutic potential beyond the genetic ablation model, we tested whether systemic, astrocyte‐targeted TSP1 delivery could prevent or reverse depressive‐like behaviors in chronic‐stress paradigms. In the chronic CORT model, a single retro‐orbital injection of AAV‐PHP.eB‐Gfap‐TSP1 at the onset of CORT exposure (P30) robustly prevented the emergence of depressive‐like behaviors typically observed after three weeks of CORT treatment (**Figure** **8**A). We further validated this approach in the CRS model,^[^ ^36^ ^]^ in which AAV‐PHP.eB‐Gfap‐TSP1 similarly reduced depressive‐like behaviors compared with EGFP controls (Figure 8B).

To test whether established phenotypes could be reversed, we administered AAV‐PHP.eB‐Gfap‐TSP1 systemically at P51, after completion of a 21‐day CORT regimen, and continued CORT exposure until P80 (Figure 8C). Under these conditions, TSP1 overexpression fully reversed behavioral deficits across all assays. Notably, repeated behavioral testing and systemic TSP1 delivery in non‐stressed WT mice did not alter baseline affective behaviors (Figure S7, Supporting Information), indicating that TSP1 does not exert nonspecific mood‐altering effects under physiological conditions. Together, these results demonstrate that systemic TSP1 delivery via AAV‐PHP.eB is sufficient to both prevent and reverse depressive‐like behaviors across multiple chronic‐stress models, supporting TSP1 as a promising astrocyte‐targeted therapeutic candidate for mood disorders.

## 3 Discussion

This study identifies astrocytic PERK deficiency as a sufficient driver of depressive‐like phenotypes. PERK (*EIF2AK3*/*Eif2ak3*) expression is reduced in PFC astrocytes from individuals with MDD and in two chronic‐stress mouse models (CORT, CRS). In otherwise healthy mice, astrocyte‐specific PERK deletion impaired intracellular Ca^2+^ signaling and suppressed expression of the synaptogenic factor TSP1 (*Thbs1*). These changes coincided with dendritic spine loss, reduced pyramidal neuron activity, diminished mPFC connectivity, and robust depressive‐like behaviors. Importantly, AAV‐mediated TSP1 restoration, delivered either locally to the mPFC or systemically via a BBB‐penetrant vector, normalized circuit measures and reversed behavioral deficits. Together, these findings indicate that astrocytic PERK signaling is essential for prefrontal circuit integrity and adaptive affective behavior, with its disruption contributing to MDD pathophysiology.

PERK is classically described as an eIF2α kinase that modulates translation during ER stress.^[^ ^22^, ^23^ ^]^ In neurons, PERK‐eIF2α signaling is tightly regulated to support long‐term synaptic plasticity and cognition,^[^ ^54^, ^55^, ^56^ ^]^ whereas chronic PERK activation can drive synaptic and cognitive decline in neurodegenerative settings.^[^ ^57^, ^58^ ^]^ Here, we extend the physiological role of PERK to astrocytes, showing that astrocytic PERK activity is required for PFC circuit regulation and affective behavior. In line with clinical relevance, *EIF2AK3* variants have been associated with depressive symptoms and neurocognitive impairment in people with HIV.^[^ ^59^, ^60^ ^]^ In young adult mice, astrocyte‐specific PERK deletion, in the absence of external stress, was sufficient to produce anxiety‐ and depressive‐like behaviors across multiple assays. Associative fear memory remained intact, in line with prior work showing preserved spatial working memory in older mice with the same manipulation,^[^ ^61^ ^]^ although broader cognitive domains warran*t* testing.

Despite PERK's canonical role in translational control, PERK deletion did not alter eIF2α protein levels or global de novo protein synthesis in astrocytes. Instead, PERK loss markedly reduced Nrf2, a transcriptional regulator of antioxidant defense.^[^ ^62^ ^]^ This observation aligns with diminished Nrf2 in the PFC of individuals with MDD^[^ ^37^ ^]^ and in stress‐susceptible rodents.^[^ ^63^ ^]^ Functionally, Nrf2 knockout mice show depressive‐like behaviors,^[^ ^64^ ^]^ whereas pharmacological Nrf2 activation confers stress resilience.^[^ ^65^ ^]^ Given Nrf2's high astrocytic expression and its role in oxidative homeostasis,^[^ ^66^, ^67^ ^]^ Nrf2 loss likely contributes to the synaptic and circuit abnormalities observed in PERK cKO mice; however, the causal relationships among Nrf2, Ca^2+^ dynamics, and TSP1 remain to be directly tested.

Astrocytic Ca^2+^ signaling is a key homeostatic regulator of neural circuits and behavior.^[^ ^68^ ^]^ PERK‐deficient astrocytes exhibited impaired intracellular Ca^2+^ dynamics, paralleling deficits reported in IP3R2 knockout mice and in models with enhanced Ca^2+^ efflux, both of which are associated with depressive‐like phenotypes.^[^ ^14^, ^18^, ^19^ ^]^ Enhancing astrocytic Ca^2+^ signaling in the PFC with Gq‐DREADDs reverses depressive‐like states in CORT‐treated mice and mice subjected to CRS,^[^ ^40^, ^69^ ^]^ and similar manipulations in the amygdala or hippocampus alleviate anxiety‐ and anhedonia‐like behaviors.^[^ ^70^, ^71^ ^]^ Mechanistically, both PERK and Nrf2 have been implicated in ER Ca^2+^ regulation,^[^ ^28^, ^29^, ^72^, ^73^ ^]^ but whether the Ca^2+^ abnormalities observed here arise directly from PERK loss, indirectly via Nrf2 downregulation, or through longer‐term astrocytic adaptations remains to be determined.

Perisynaptic astrocytic Ca^2+^ transients shape synaptic plasticity through gliotransmission and release of synaptogenic factors.^[^ ^74^, ^75^, ^76^ ^]^ In PERK cKO mice, reduced perisynaptic Ca^2+^ activity coincided with increased spine elimination, decreased levels of PSD95 and Homer1, and reduced pyramidal neuron firing and connectivity. These convergent alterations support a model in which PERK loss weakens astrocyte‐neuronal signaling, leading to structural and functional synaptic deficits. Future electrophysiological studies will be important for defining how PERK signaling tunes astrocyte‐mediated modulation of synaptic transmission and to test whether changes in synaptic efficacy track the structural and activity shifts observed here.

Our data implicate TSP1 as a key downstream effector linking astrocytic PERK deficiency to synaptic and circuit dysfunction. *Thbs1*/*THBS1* expression was reduced in PERK cKO mice, in CORT‐treated mice, and in PFC astrocytes from individuals with MDD, paralleling decreased serum TSP1 reported in depression.^[^ ^77^ ^]^ TSP1, secreted by cortical astrocytes, promotes excitatory synaptogenesis and spine maturation via α_2_δ‐1 subunits of neuronal voltage‐gated calcium channels,^[^ ^32^, ^78^ ^]^ and its transcription and release are Ca^2+^ dependent.^[^ ^51^, ^79^, ^80^ ^]^ These observations support a framework in which PERK sustains astrocytic Ca^2+^ dynamics that maintain TSP1 expression and availability, stabilizing excitatory synapses and supporting mPFC function. Reduced pyramidal neuron activity and connectivity in PERK cKO mice may therefore reflect combined effects of spine loss and diminished astrocyte‐derived trophic support. Although our experiments emphasize excitatory synapses, future studies should incorporate inhibitory markers (e.g., gephyrin/VGAT), test whether acute TSP1 release is sufficient to normalize circuit function, and examine the roles of other thrombospondins that may be differentially affected by PERK loss.

Therapeutically, TSP1 restoration was sufficient to rescue synaptic density, enhance mPFC neuronal activity and functional connectivity, and reverse depressive‐like behaviors across PERK cKO, CORT, and CRS models. Systemic delivery of a BBB‐penetrant vector achieved broad astrocytic transduction without evidence of BBB disruption, underscoring the translational promise of astrocyte‐targeted TSP1 augmentation.

Several limitations merit consideration. First, human RNAscope analyses were performed on postmortem tissue from relatively older individuals; transcriptomic profiling in younger cohorts will be important to clarify the role of PERK across the lifespan in MDD. Second, apart from the astrocyte‐specific PERK cKO, we focused primarily on CORT and CRS paradigms, which do not capture the full complexity of MDD, including psychosocial stressors.^[^ ^81^ ^]^ Additional models (e.g., chronic social defeat stress, chronic unpredictable mild stress) may provide complementary insights. Third, although PERK deletion produced Nrf2 downregulation, Ca^2+^ deficits, and TSP1 suppression, the causal ordering among these events remains unresolved. Direct manipulation of Nrf2, with concurrent measurements of Ca^2+^ dynamics and TSP1 secretion, will be necessary to delineate this pathway more precisely.

In summary, astrocytic PERK emerges as a critical regulator of prefrontal circuitry and affective behavior, acting through coordinated changes in Nrf2, Ca^2+^ signaling, and TSP1. The convergence of reduced PERK, Nrf2, and TSP1 in PFC astrocytes from individuals with MDD underscores the clinical relevance of this pathway. Targeted restoration of astrocytic TSP1, particularly via BBB‐penetrant AAV, represents a promising strategy to reestablish prefrontal circuit function and enhance stress resilience in depression.

## 4 Experimental Section

### Human Postmortem Brain Tissue

Postmortem human brain tissue was obtained from the NIH NeuroBioBank (via participating repositories) and the New York Brain Bank. All specimens were de‐identified prior to distribution. Informed consent for donation was obtained by the repositories from donors or next of kin. Specimen metadata are provided in Table S1 (Supporting Information). All procedures were approved by the Institutional Review Board (IRB).

### Animals

All animal procedures were approved by the Institutional Animal Care and Use Committee (IACUC) at Columbia University (protocol AABN7553) and conformed to the NIH Guide for the Care and Use of Laboratory Animals. Mouse strains included the following from The Jackson Laboratory: C57BL/6J (JAX #000 664), Aldh1l1‐Cre/ER^T2^ (JAX #029 655), PERK^loxP^ (JAX #023 066), Thy1‐YFP‐H (JAX #003 782), and Ai14 (JAX #007 914). Aldh1l1^CreERT2/+^;PERK^fl/fl^ mice were generated by crossing Aldh1l1‐Cre/ER^T2^ with PERK^loxP^ mice. Thy1‐GCaMP6s (founder line 1) mice were bred in‐house.^[^ ^82^ ^]^

Mice were group‐housed in temperature‐ and humidity‐controlled rooms on a 12‐h light/dark cycle with ad libitum access to food and water. For PERK knockout experiments, mice of both sexes (1–3 months old) were used. For chronic corticosterone (CORT) and chronic restraint stress (CRS) paradigms, only male mice (1–2 months old) were used.

To induce astrocytic PERK deletion, Aldh1l1^CreERT2/+^;PERK^fl/fl^ mice received 4‐hydroxytamoxifen (4‐OHT; 50 mg kg^−1^, Sigma–Aldrich H6278) intraperitoneally once daily for three consecutive days; PERK^fl/fl^ littermate controls received identical 4‐OHT treatments. For chronic CORT exposure, mice received corticosterone (0.1 mg mL^−1^) in the drinking water for three consecutive weeks.^[^ ^8^, ^35^ ^]^ For CRS, mice were placed in ventilated 50‐mL conical tubes for 2–4 h daily over 14 consecutive days, which is a widely used and validated model of depression‐like behavior.^[^ ^36^, ^83^ ^]^

### Plasmids and Vectors

The pAAV‐gfaABC1D‐G‐CEPIA1er plasmid^[^ ^41^ ^]^ (generously provided by Dr. Yohei Okubu) was used to generate AAV5‐gfaABC1D‐G‐CEPIA1er (3.52 × 10^13^ genome copies [GC]/mL; OriGene Technologies). AAV5‐gfaABC1D‐cyto‐GCaMP6f (Addgene #52 925), AAV5‐gfaABC1D‐Lck‐GCaMP6f (Addgene #52 924), and AAV5‐gfaABC1D‐tdTomato (Addgene #44 332) were obtained from Addgene. The pAAV‐Gfap‐TSP1‐HA and pAAV‐Gfap‐EGFP plasmids^[^ ^84^ ^]^ were kindly provided by Drs. Kevin K. Park and Konstantin Levay. The following vectors were produced by Virovek, Inc.: AAV5‐Gfap‐TSP1‐HA (1.0 × 10^13^ GC mL^−1^), AAV5‐Gfap‐EGFP (1.0 × 10^13^ GC mL^−1^), AAV‐PHP.eB‐Gfap‐TSP1‐HA (1.0 × 10^13^ GC mL^−1^), and AAV‐PHP.eB‐Gfap‐EGFP (2.0 × 10^13^ GC mL^−1^). AAV5‐GFAP‐mCherry‐Cre was obtained from the University of North Carolina Vector Core.

For stereotaxic injection into the medial prefrontal cortex (mPFC), AAVs were delivered at the following coordinates relative to bregma: anteroposterior (AP) +2 mm, mediolateral (ML) +0.4 mm, and depth –0.8 mm from the pial surface. Injections were performed using a picospritzer (5 psi, 12 ms pulse width, 0.5 Hz) through a pulled glass micropipette. To minimize reflux, the micropipette was left in place for 10 min after infusion before withdrawal. For broad CNS transduction, AAV‐PHP.eB vectors were administered via retro‐orbital injection (1 × 10^12^ GC mL^−1^; 100 µL).

### Immunofluorescence and In Situ Hybridization

Mice were deeply anesthetized and transcardially perfused with phosphate‐buffered saline (PBS) followed by 4% paraformaldehyde (PFA). Brains were postfixed overnight at 4 °C in 4% PFA, cryoprotected in 30% sucrose for 48 h, and sectioned at 60 µm using a Leica VT‐1000S vibratome. Free‐floating sections were rinsed twice in PBS, then permeabilized and blocked for 2 h at room temperature in PBS containing 0.4% Triton X‐100 and 1% donkey serum.

For immunofluorescence, sections were incubated with primary antibodies (Table S2, Supporting Information) for 48 h at 4 °C, washed in PBS, and then incubated with Alexa Fluor‐conjugated secondary antibodies for 1 h at room temperature. After final PBS washes, sections were mounted in DAPI‐containing medium for confocal imaging.

For mouse or human RNAscope FISH, fresh‐frozen brain sections (20 µm) were mounted on glass slides. Tissue pretreatment and fluorescent multiplex assays were performed according to the manufacturer's instructions (Advanced Cell Diagnostics). RNA probes (Table S3, Supporting Information) targeted mouse *S100β*, *Aldh1l1*, *Eif2ak3*, and *Thbs1*, and human *GFAP*, *EIF2AK3*, and *THBS1*, and were visualized with fluorophore‐conjugated labels.

Images were acquired on a Nikon Ti‐based laser‐scanning confocal microscope using 20× and 63× objectives. Immunofluorescence images were collected at 512 × 512 pixels; RNAscope and synaptic‐marker images were collected at 1024 × 1024 pixels. Paired images were captured under identical settings and processed uniformly. Image analysis was performed in ImageJ (NIH).

For RNAscope FISH analysis, fields of view (FOVs) were selected using systematic uniform random sampling with a randomized grid origin. Within each FOV, marker‐verified astrocytes were selected by nucleus‐based random sampling. This yielded 16–36 astrocytes per case across 4–6 sections, with 4–6 FOVs per section. *Eif2ak3* and *Thbs1* puncta were quantified using a fixed spot‐detection pipeline with uniform thresholds by raters blinded to group assignment. Cell‐level measurements were averaged to a single value per case for statistical analysis.

### Astrocyte Isolation

Adult astrocytes were isolated by magnetic‐activated cell sorting (MACS; Miltenyi Biotec, 130‐097‐678). Mice were deeply anesthetized and transcardially perfused with PBS. After meningeal removal, the mPFC was dissected and dissociated into single cells using the Adult Brain Dissociation Kit (Miltenyi Biotec, 130‐107‐677) in C‐tubes, followed by mechanical processing on a gentleMACS Dissociator (Miltenyi Biotec, 130‐096‐427). Cell suspensions were centrifuged (300 × g, 3 min, 4 °C), passed through a 70‐µm strainer (Miltenyi Biotec, 130‐098‐462), washed with PBS, and resuspended in PBS. Suspensions were incubated with anti‐myelin microbeads (15 µL; 10 min, 4 °C) with gentle mixing every 5 min, and then applied to an LS column on a MACS Separator. The flow‐through was collected, centrifuged, and resuspended in PBS. Cells were blocked using FcR blocking reagent (15 µL; 10 min, 4 °C) and labeled with anti‐ACSA‐2 microbeads (15 µL; Miltenyi Biotec ACSA‐2 kit; 10 min, 4 °C). After removing unbound beads, the suspension was applied to an LC column on the MACS Separator to enrich ACSA‐2^+^ astrocytes. The astrocyte‐enriched fraction was collected, centrifuged, snap‐frozen in liquid nitrogen, and stored at –80 °C until RT‐qPCR or immunoblotting. Sorted fractions were evaluated by immunoblotting for TUJ1 (neuronal marker), which showed negligible signal (Figure S1C, Supporting Information), indicating high astrocyte purity.

### Immunoblotting

Isolated astrocytes or bulk mPFC tissue were sonicated in ice‐cold lysis buffer supplemented with protease and phosphatase inhibitor cocktails (Thermo Scientific, 78 442). Lysates were clarified by centrifugation (12 000 × g, 10 min, 4 °C). Supernatants were collected. Protein concentrations were determined using a BCA assay (Thermo Scientific, 23 227). Equal amounts of protein were resolved by SDS‐PAGE on 8–12% Bis‐Tris polyacrylamide gels in Bio‐Rad running buffer for ≈1 h and then transferred to PVDF or nitrocellulose membranes using the Bio‐Rad transfer system. Membranes were blocked, incubated overnight at 4 °C with primary antibodies (Table S2, Supporting Information), washed twice in PBST (PBS containing 0.1% Tween‐20), and then incubated with HRP‐conjugated secondary antibodies (anti‐rabbit IgG, CST 7074S; anti‐mouse IgG, CST 7076S) for 1 h at room temperature. Protein bands were visualized using an ImageQuant LAS‐4000 system and quantified with ImageQuant software.

### Measurement of Protein Synthesis

De novo protein synthesis was quantified using the Surface Sensing of Translation (SUnSET) assay,^[^ ^85^ ^]^ which detects puromycin incorporation into nascent peptides.^[^ ^86^ ^]^ To restrict the analysis to astrocytes, 0.2 µL of AAV5‐gfaABC1D‐tdTomato was stereotaxically injected into the mPFC of 1‐month‐old mice. At 2 months of age, mice were implanted with an intracranial cannula targeting the mPFC. Puromycin (5 µg; Sigma, P8833) was infused via the cannula using a Hamilton syringe connected to an infusion pump. After a 1‐h incorporation period, brains were collected and processed for immunofluorescent detection of puromycin‐labeled proteins within tdTomato^+^ astrocytes.

### Surgical Preparation for In Vivo Imaging

To enable cellular and subcellular imaging of the mPFC, a 1.0‐mm microprism (Tower Optical, 4531‐0022) was implanted in the contralateral hemisphere following established protocols.^[^ ^8^, ^87^, ^88^ ^]^ Mice were anesthetized with ketamine (100 mg kg^−1^) and xylazine (15 mg kg^−1^). After a midline scalp incision, the skull was exposed, and a custom head bar was affixed. An ≈2‐mm circular craniotomy was made over the mPFC (AP +2 mm; ML +0.4 mm) using a high‐speed dental drill, taking care to avoid brain compression and the superior sagittal sinus. The dura adjacent to the sinus was carefully removed. A microprism, pre‐mounted to a coverslip with UV‐curable optical adhesive, was slowly inserted between the hemispheres over 5–10 min and aligned to the midline. The gap between the coverslip and skull was sealed with tissue adhesive, and the prism‐coverslip assembly was secured with dental cement. After curing, a thin silicone layer was applied to protect the window.

Mice were maintained on a heating pad (≈37 °C) throughout surgery and recovery. Postoperatively, animals received meloxicam and sulfamethoxazole, and were returned to their home cages. Consistent with prior reports,^[^ ^8^, ^88^ ^]^ prism implantation did not induce detectable gliosis in the mPFC (Figure S3A, Supporting Information); at 2 weeks post‐surgery, GFAP^+^ astrocyte counts were comparable to non‐surgical controls. Imaging began 4 weeks after implantation, with a 20‐min head restraint habituation on the imaging platform before each session.

### In Vivo Ca^2+^ Imaging and Data Analysis

Astrocytic and neuronal Ca^2+^ dynamics were monitored using genetically encoded Ca^2+^ indicators: G‐CEPIA1er for endoplasmic reticulum (ER) Ca^2+^ signals^[^ ^41^ ^]^ and GCaMP6 for cytosolic signals.^[^ ^89^ ^]^ For astrocytic ER Ca^2+^ imaging, 0.3 µL of a 1:1 mixture of AAV5‐gfaABC1D‐G‐CEPIA1er and AAV5‐gfaABC1D‐tdTomato (an astrocyte soma marker) was injected into the prelimbic mPFC. For astrocytic cytosolic Ca^2+^ imaging, 0.3 µL of AAV5‐gfaABC1D‐cyto‐GCaMP6f was injected into the mPFC. To monitor perisynaptic astrocytic Ca^2+^ near dendritic spines, 0.3 µL of a 1:10 000 mixture of AAV5‐gfaABC1D‐cyto‐GCaMP6f and AAV5‐phSyn‐tdTomato was injected to achieve sparse dendritic labeling (2–5 segments per FOV). Neuronal Ca^2+^ imaging was performed in Thy1‐GCaMP6s mice, which express GCaMP6s in layer 5 pyramidal neurons.

In vivo imaging was performed using a Scientifica two‐photon microscope equipped with a Ti:sapphire laser (Chameleon Vision‐S, Coherent) tuned to 920 nm and a 25×/1.05 NA objective with artificial cerebrospinal fluid as the immersion medium. Astrocyte somata were imaged 100–300 µm below the pial surface; neuronal somata (layer 5) at 500–700 µm; and apical dendrites at 50–100 µm. Digital zoom settings were ×2 (astrocyte somata), ×4 (astrocyte processes), ×1.5 (neuronal somata), ×2 (neuronal dendrites). Images were acquired at 1.07 Hz (512 × 512 pixels; 2 µs pixel dwell) with ScanImage v5.4 (Vidrio Technologies). Sessions with excessive motion artifacts were excluded. Where indicated, a 2‐s air puff (0.1‐mm PVC tubing positioned ∼2 cm from the nostril) was delivered to elicit ER Ca^2+^ release.

Images were processed in ImageJ.^[^ ^90^ ^]^ Motion correction was performed using TurboReg. Regions of interest (ROIs) were manually drawn for astrocyte somata and processes, neuronal somata, and dendrites. For perisynaptic analyses, circular ROIs (radius of 2.5 µm) were centered on dendritic spine heads to capture adjacent astrocytic signals. Background‐subtracted mean fluorescence per ROI was expressed as Δ*F*/*F*_0_ = (*F* − *F*_0_)/*F*_0_, where *F*_0_ was the mean of the lowest 6‐s interval within each 5‐min recording. Ca^2+^ transients were defined as events >3 standard deviations above baseline. Quantified metrics included frequency (events per minute), amplitude (peak Δ*F*/*F*_0_), and area under the curve (AUC; Δ*F*/*F*_0_ per minute). Neuronal functional connectivity was computed from Pearson's correlation coefficients (*r*) between Ca^2+^ transient time series for all active neuron pairs and visualized in RStudio with the *corrplot* package. For statistical analyses, the mean *r* per animal was calculated and used for group comparisons.

### Fiber Photometry

A 400‐µm outer diameter, 0.48‐NA optical fiber mounted in a ceramic ferrule (Doric Lenses) was stereotaxically implanted into the mPFC of Thy1‐GCaMP6s mice. Animals recovered for at least 4 weeks before recording sessions. During recording, excitation light from a 470‐nm LED (modulated at 521 Hz) passed through an excitation filter, was reflected by a dichroic mirror, and was coupled into the implanted fiber. Emitted fluorescence was collected through the same fiber, separated by the dichroic mirror, detected by a photodetector, demodulated by lock‐in detection, and low‐pass filtered. Fluorescence was normalized within each trial as Δ*F*/*F*_0_, where *F*_0_ was the mean baseline during the pre‐struggle phase of the tail suspension test. After recordings, mice were perfused and the brains were examined to confirm fiber placement in the mPFC.

### Dendritic Spine Imaging and Data Analysis

Dendritic spine dynamics in the mPFC were tracked over a 10‐day period in Thy1‐YFP‐H mice.^[^ ^90^ ^]^ Imaging was performed through the implanted microprism using a two‐photon microscope (920‐nm excitation) equipped with a 60×/1.1 NA water‐immersion objective. 3D image stacks of dendrites within 100 µm of the pial surface were acquired at a resolution of 1024 × 1024 pixels; 4–6 stacks were collected per session for each animal. Between sessions, mice were returned to their home cages.

Image analysis was performed in ImageJ. Filopodia were defined as long, thin protrusions with a head‐to‐neck diameter ratio of <1.2 and a length‐to‐neck diameter ratio of >3; all other protrusions were classified as dendritic spines. Spines were considered stable if their positions remained unchanged relative to nearby structural landmarks across sessions. Spines were classified as newly formed or eliminated if their positions shifted by more than 0.7 µm from the original location. For each mouse, at least 150 spines were analyzed. Formation and elimination rates were calculated as the number of spines formed or eliminated between sessions divided by the total number of spines present at the initial session.

### Bulk RNA Sequencing

Total RNA was extracted from the mPFC using the Total RNA Mini Kit (Bio‐Rad, 7 326 820). RNA integrity was assessed using an Agilent Bioanalyzer; only samples with RIN ≥ 9.5 were advanced for sequencing. Library preparation and sequencing were performed at the Columbia Genome Center using a standard stranded poly(A) RNA‐seq workflow. Briefly, mRNA was enriched by poly(A) selection, libraries were prepared with Illumina TruSeq chemistry, and sequencing was performed on an Illumina NovaSeq 6000. Raw reads were pseudoaligned to the mouse transcriptome (GRCm38) with *kallisto*. Differential gene expression was analyzed with *DESeq2*, and gene ontology (GO) enrichment was performed using enrichGO (*clusterProfiler*) in RStudio.

### RT‐PCR

Total RNA from bulk mPFC tissue or isolated astrocytes was reverse‐transcribed into cDNA using SuperScript IV VILO Master Mix (Thermo Fisher, 1 175 650). Quantitative real‐time PCR (RT‐qPCR) was performed on a QuantStudio Real‐Time PCR System (Thermo Fisher) using SYBR Green PCR Master Mix (Thermo Fisher, 4 309 155). Each sample was run in duplicate or more. Primer sequences were validated and are listed in Table S4 (Supporting Information).

### Blood‐Brain Barrier Permeability

To assess whether AAV‐PHP.eB‐Gfap‐TSP1 affects blood‐brain barrier (BBB) integrity, mice received a retro‐orbital injection of a 70‐kDa rhodamine‐dextran (0.2 mL). Two‐photon image stacks of cortical parenchyma were acquired with 830‐nm excitation, at depths of 50–300 µm below the pial surface. BBB permeability was evaluated by comparing extravascular fluorescence immediately before and 20 min after injection, with all imaging parameters held constant across animals.

### Behavior Analysis

Experimental groups were randomly assigned. All behavioral testing was conducted with experimenters blinded to group identity until data collection was complete. For cohorts run on multiple assays, tests were administered in order from least to most stressful, with 24‐h intervals: open field, elevated plus maze, sucrose preference, tail suspension, and forced swim. Mice were acclimated to the behavior room for at least 60 min before each test.

*Open field test*: General locomotion and anxiety‐like behavior were assessed in a dimly lit 40 cm × 40 cm arena. Each mouse was placed in the center and was allowed to explore freely for 6 min. Anxiety‐like behavior was quantified as the amount of time spent in the central 20 × 20 cm zone. Total distance and center time were recorded using ANY‐maze (Stoelting). For locomotor effects of AAV‐PHP.eB‐Gfap‐TSP1, a separate 20‐min session was conducted, and total distance was analyzed in 2‐min bins.

*Elevated plus maze*: The apparatus comprised a central platform with two open and two closed arms, elevated 50 cm above the floor. Mice explored for 6 min beginning from the center of the platform. Time spent in open arms was recorded and analyzed using ANY‐maze.

*Forced swim test*: Mice were placed individually in cylindrical glass beakers (19‐cm diameter × 15‐cm height) filled with 10 cm of water at 23 ± 2 °C. Behavior was recorded for 6 min; immobility was quantified during the final 4 min. Immobility was defined as passive floating with only minimal movement to keep the head above water.

*Tail suspension test*: Mice were suspended by the tail with adhesive tape attached to a nylon string, with no contact with nearby surfaces. Behavior was recorded for 6 min; immobility during the final 4 min was quantified using ANY‐maze. Immobility was defined as the absence of limb or body movements aside from respiration or minimal postural adjustments.

*Sucrose preference test*: Mice were single‐housed and water‐deprived for 8 h before testing. Each mouse had simultaneous access to two bottles (water vs 1.5% sucrose). After 4 h, bottle positions were swapped to control for side bias. Sucrose preference was calculated as the sucrose volume consumed divided by the total fluid intake (sucrose + water).

*Fear conditioning*: Fear conditioning was conducted in a conditioning chamber (Maze Engineers) with ANY‐maze recording. On training day, mice were habituated for 2 min, followed by three pairings of a 30‐s auditory cue (400 Hz, 80 dB) co‐terminated with a 2‐s foot shock (0.5 mA), with 30‐s intertrial intervals; mice were returned to their home cages 2 min after the final pairing. Cued recall was assessed 24 h later in a distinct context with the same auditory cue. Freezing behavior was quantified automatically using ANY‐maze; auditory‐cued memory was defined as the percentage of time spent freezing during the tone.

*Von Frey tests*: Mechanical sensitivity was measured using calibrated von Frey filaments (0.07–2.0 g) applied to the plantar surface of the hind paw. Mice were placed in transparent acrylic chambers on an elevated mesh platform and habituated for 30 min. Paw‐withdrawal thresholds were determined using the up‐down (Dixon) method.

### Statistical Analysis

Data are presented as mean ± SEM. No formal statistical methods were used to predetermine sample sizes; however, the sizes selected for in vivo imaging and behavioral assays were consistent with those reported in similar studies.^[^ ^91^, ^92^ ^]^ No successfully imaged or measured samples or animals were excluded from the analysis. Data normality was assessed with the Shapiro‐Wilk test. For normally distributed data, parametric tests were used; for non‐normal data, nonparametric tests were applied. Two‐group comparisons used unpaired *t* tests or Mann‐Whitney U tests, as appropriate. For analyses involving more than two groups, one‐way or two‐way ANOVA was performed followed by Bonferroni post hoc tests. All tests were two‐sided, with significance set at *P* ≤ 0.05. Analyses were conducted in GraphPad Prism version 8.4. Additional statistical details are provided in the figure legends.

## Conflict of Interest

The authors declare no conflict of interest.

## Author Contributions

Conceptualization by K.C. and G.Y.; Methodology by K.C., Y.M., Y.Q., X.D., C.P., O.A.‐D., M.B.D., and G.Y.; Investigation by K.C., R.G., and Y.M.; Visualization by K.C. and G.Y.; Funding acquisition by G.Y.; Supervision by G.Y.; Writing – original draft by K.C. and G.Y.; Writing – review & editing by K.C., R.G., Y.M., Y.Q., X.D., C.P., O.A.‐D., M.B.D., and G.Y.; All authors read and approved the final manuscript.

## Supporting information



Supporting Information

Supplemental Video 1

## Data Availability

Data associated with this article are depoisted in the Dryad Repository (https://doi.org/10.5061/dryad.mkkwh71dq).
