# Supplementary material for: Astrocytic PERK Deficiency Drives Prefrontal Circuit Dysfunction and Depressive‐Like Behaviors
Source: Adv Sci (Weinh). 2025 Nov 30;13(9):e10780. doi: 10.1002/advs.202510780 (PMC12904067; doi:10.1002/advs.202510780)
Supplement: Supplementary file 1 — Supporting Information [file ADVS-13-e10780-s002.pdf]

Supporting Information

Astrocytic PERK Deficiency Drives Prefrontal Circuit Dysfunction and Depressive-Like Behaviors

Kai Chen, Riya Gupta, Yosuke M. Morizawa, Yu Qin, Xingyu Du, Cynthia Pang,
Osama Al-Dalahmah, Maura B. Dupont, Guang Yang

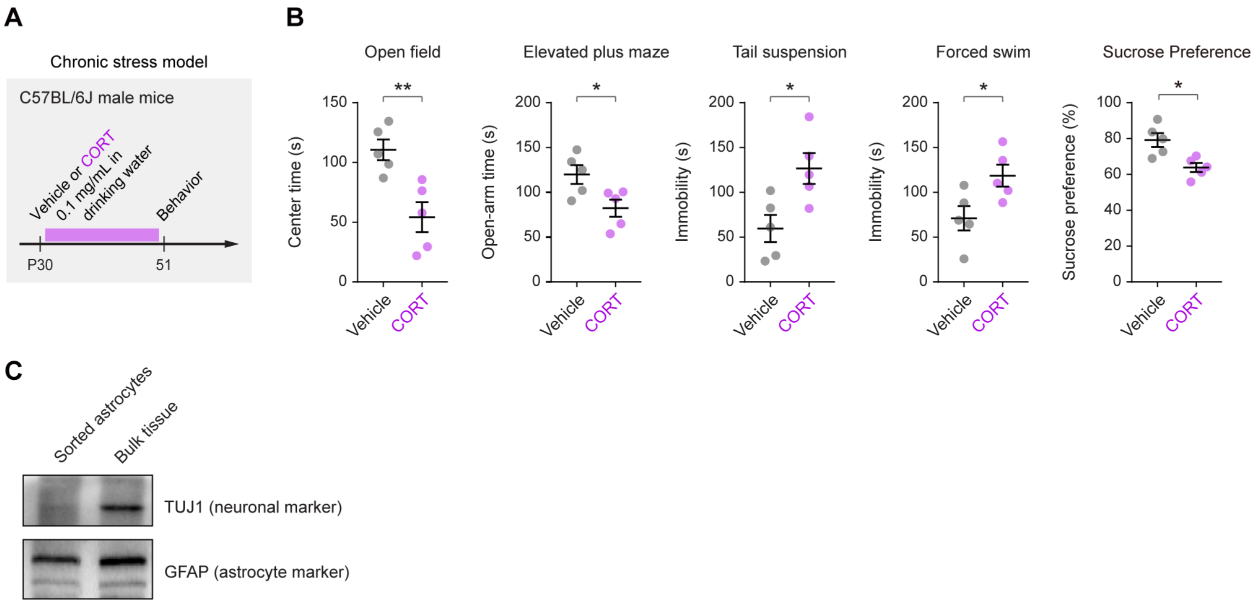

Figure S1. Chronic CORT exposure induces anxiety- and depressive-like behaviors in mice

(**A**) Experimental timeline for chronic corticosterone (CORT) administration. (**B**) Behavioral performance of vehicle- and CORT-treated mice (n = 5 mice/group) in the open field (t_8 = 3.697, P = 0.0061), elevated plus maze (t_8 = 2.648, P = 0.0294), tail suspension test (t_8 = 2.927, P = 0.0191), forced swim test (t_8 = 2.590, P = 0.0321), and sucrose preference test (t_8 = 3.306, P = 0.0108), demonstrating that chronic CORT exposure induces robust anxiety- and depressive-like behaviors. (**C**) Representative immunoblot for TUJ1 (neuronal marker) in MACS-isolated astrocyte fractions demonstrates minimal signal, indicating high astrocyte purity. Summary data are mean ± SEM. *P < 0.05, **P < 0.01; two-tailed unpaired t test. Each dot represents one mouse.

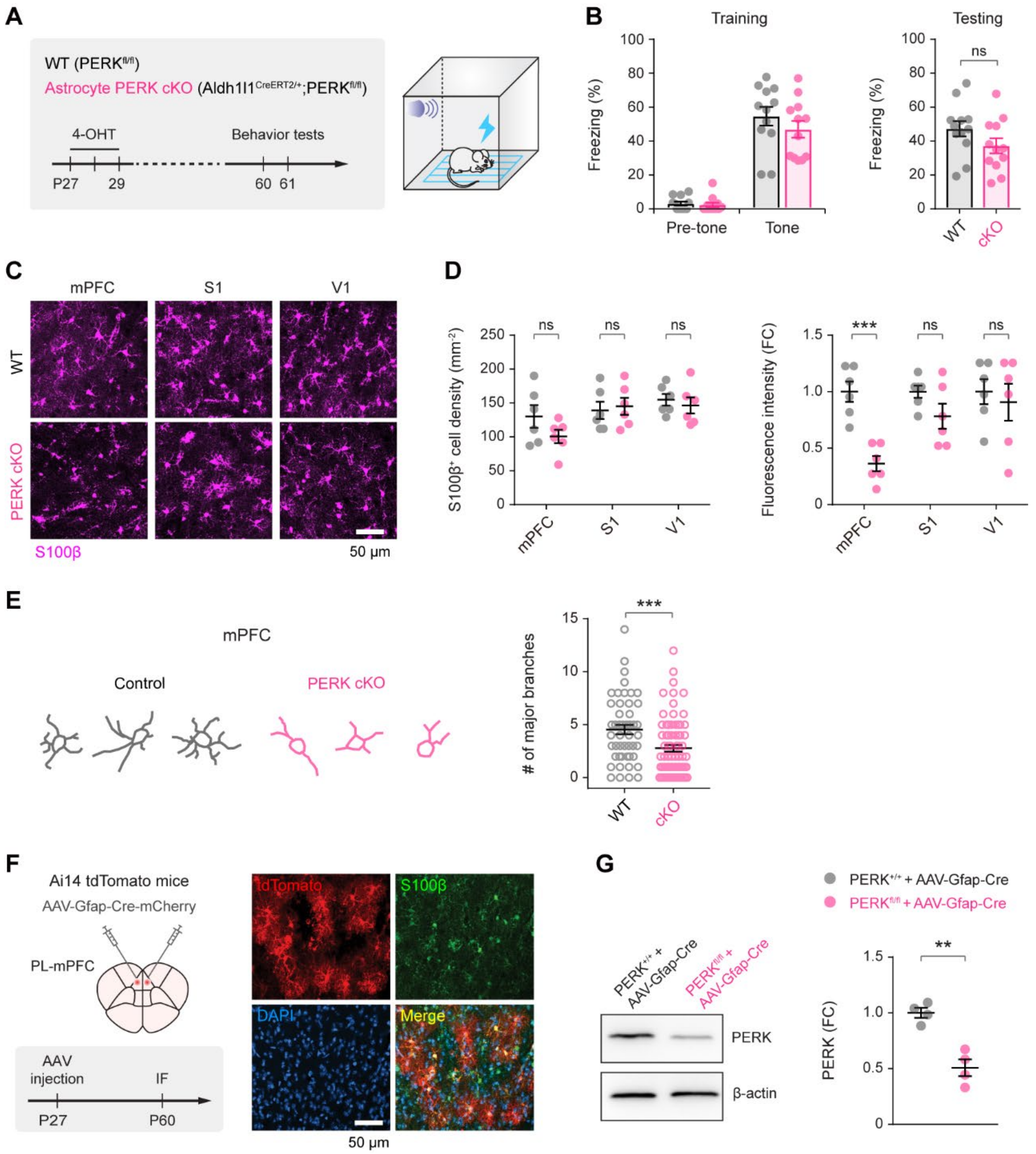

Figure S2. Effects of astrocytic PERK deletion on associative fear memory and mPFC astrocytes

(**A**) Experimental timeline for the auditory-cued fear conditioning paradigm. (**B**) Left, percentage freezing before and after conditioning on the training day (*n* = 12 mice/group). Right, auditory-cued freezing 24 h after training. Astrocyte-specific PERK deletion does not impair auditory-cued associative fear memory (t_{22} = 1.601, *P* = 0.1236; two-tailed unpaired t test). (**C**) Representative immunofluorescence images showing S100β$^+$ astrocytes in the medial prefrontal cortex (mPFC), primary somatosensory cortex (S1), and primary visual cortex (V1) of WT and PERK cKO mice. (**D**) Quantification of S100β$^+$ cell density and fluorescence intensity in these regions (*n* = 6 mice/group). Cell density did not differ between genotypes in mPFC, S1, or V1 (*P*

= 0.2944, >0.9999, >0.9999), whereas fluorescence intensity was selectively reduced in the mPFC but not in S1 or V1 (P = 0.0006, 0.4726, >0.9999; two-way ANOVA followed by Bonferroni's test). (**E**) Left, schematic of astrocyte branching analysis. Right, PERK cKO mice exhibit fewer branches per S100β$^+$ astrocyte in the mPFC (n = 50–75 cells from 5 mice/group; U = 1198, P = 0.0005, Mann-Whitney U test). (**F**) Left, experimental timeline for validation of astrocyte-specific Cre expression using AAV-Gfap-Cre in the Ai14 reporter line. Right, representative images showing tdTomato$^+$ cells co-labeled with S100β in the mPFC. (**G**) Immunoblotting of sorted mPFC astrocytes confirms AAV-mediated PERK deletion in PERK$^{fl/fl}$ mice (n = 4 mice/group; t_6 = 5.641, P = 0.0013; two-tailed unpaired t test). Summary data are mean ± SEM. ns, not significant; **P < 0.01, ***P < 0.001. Each dot represents one mouse (**B**, **D**, and **G**) or one astrocyte (**E**). FC, fold change.

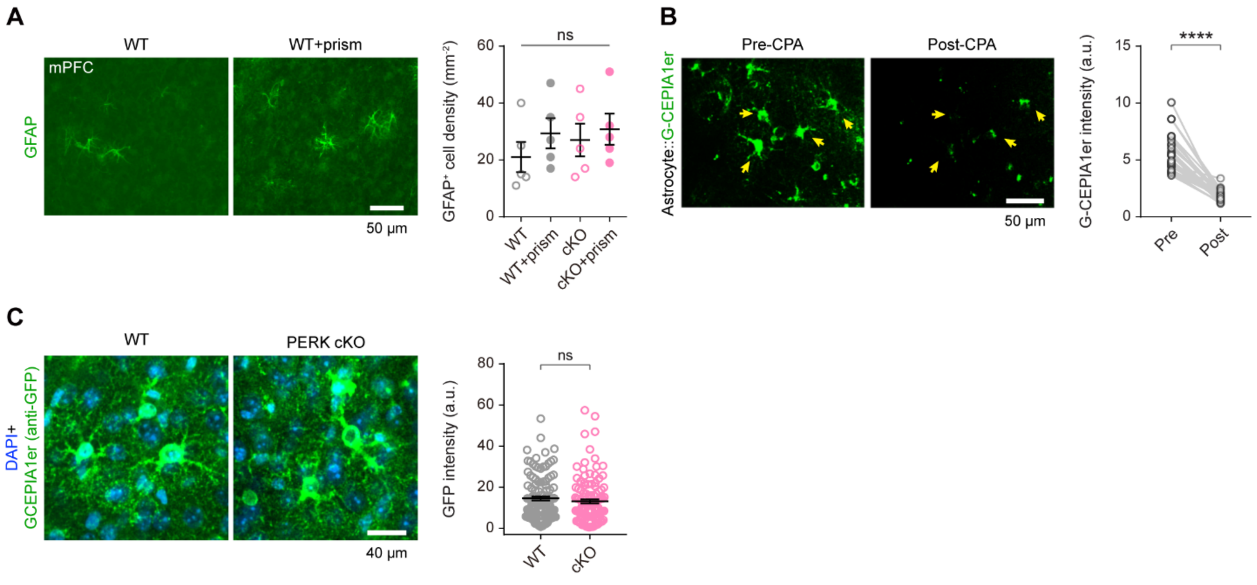

Figure S3. Characterization of mPFC astrocytes expressing the ER Ca^{2+} sensor G-CEPIA1er

(**A**) Left, representative confocal images of mPFC sections immunostained for GFAP in mice with or without chronic microprism implantation. Right, $GFAP^+$ astrocyte density did not differ between groups (*n* = 5 mice/group; two-way ANOVA, main effect of implantation: $F_{1, 16}$ = 1.239, *P* = 0.2821), indicating that microprism implantation does not induce astrogliosis in the mPFC. (**B**) Left, two-photon images of mPFC astrocytes expressing G-CEPIA1er before and after local infusion of cyclopiazonic acid (CPA, 50 µM), an inhibitor of sarco/endoplasmic reticulum Ca^{2+}-ATPase (SERCA). Right, G-CEPIA1er fluorescence was significantly decreased after CPA (*n* = 30 cells from 3 mice; t_{29} = 13.03, *P* < 0.0001; two-tailed paired t test), confirming the responsiveness of the sensor to ER Ca^{2+} depletion. (**C**) Left, representative cortical sections showing AAV-mediated expression of G-CEPIA1er (anti-GFP immunostaining) in mPFC astrocytes. Right, baseline G-CEPIA1er signal did not differ between WT and PERK cKO mice (*U* = 6322, *P* = 0.1856; Mann-Whitney U test). Summary data are mean ± SEM. ns, not significant; *****P* < 0.0001. a.u., arbitrary units.

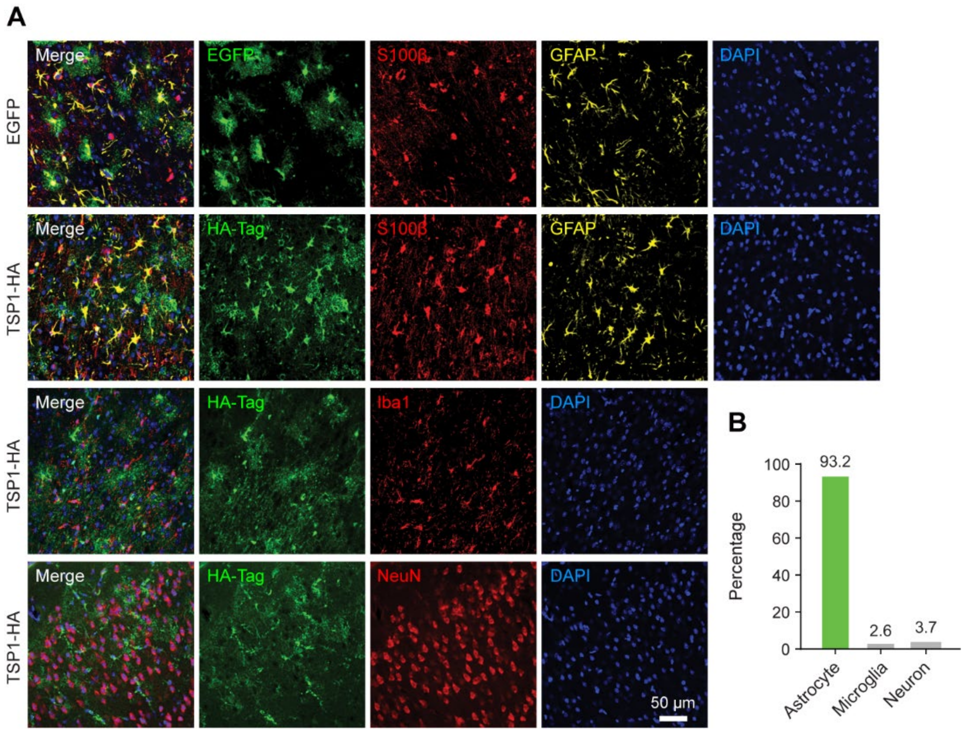

Figure S4. Characterization of AAV-mediated gene delivery to mPFC astrocytes

(**A**) Representative confocal images of the mPFC following infection with AAV5-Gfap-TSP1-HA or AAV5-Gfap-EGFP (control). Sections were co-immunostained for astrocytic markers (S100β, GFAP), the microglial marker (Iba1), and the neuronal marker (NeuN) to assess cell-type-specific transduction. (**B**) Quantification of the cell-type specificity of AAV5-Gfap-TSP1-HA expression. Approximately 93% of TSP1-HA-expressing (HA^+) cells are astrocytes ($S100\beta^+$ and/or $GFAP^+$), with only rare labeling of $Iba1^+$ microglia or $NeuN^+$ neurons (n = 4 mice).

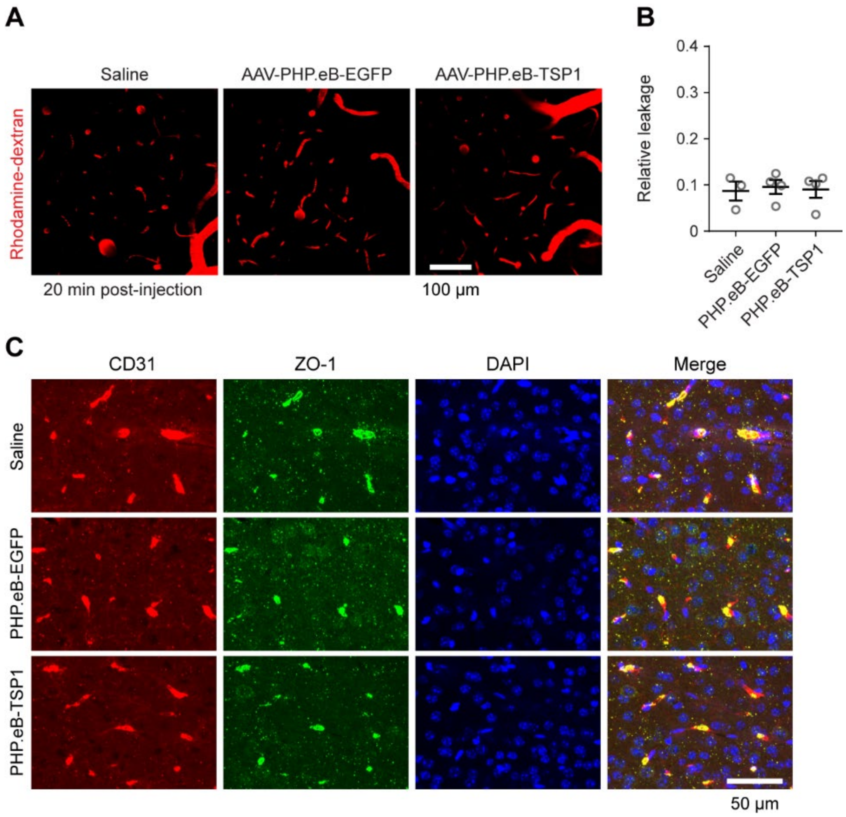

Figure S5. Systemic AAV-PHP.eB delivery does not compromise blood-brain barrier (BBB) integrity

(**A**) Representative in vivo two-photon images of cortical vasculature obtained following intravenous injection of rhodamine-dextran under the indicated treatment conditions. (**B**) Vascular leakage was quantified as the ratio of extravascular fluorescence at 20 min post-injection relative to baseline (pre-injection). No significant differences in leakage were detected among treatment groups (n = 3–4 mice/group; one-way ANOVA, $F_{2, 8} = 0.06608$, $P = 0.9366$). (**C**) Representative confocal sections stained for CD31 (endothelial marker) and ZO-1 (tight-junction marker) show robust colocalization along vessel walls, indicating preserved BBB integrity following systemic AAV-PHP.eB administration. Summary data are mean ± SEM. Each dot represents one mouse.

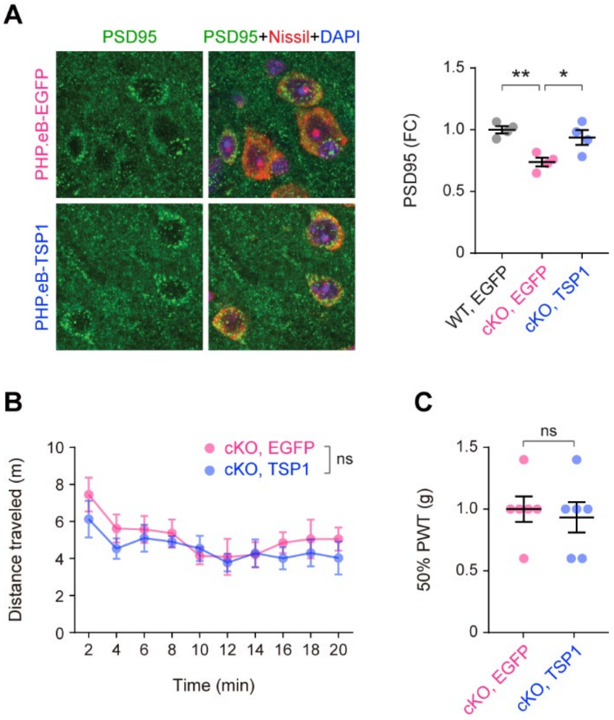

Figure S6. AAV-PHP.eB-mediated astrocytic TSP1 expression enhances synaptic markers without affecting locomotion or mechanosensation in PERK cKO mice

(**A**) Immunostaining and quantification of PSD95 puncta in the mPFC (n = 4 mice/group; P = 0.0066, 0.0305; one-way ANOVA with Bonferroni's post hoc test). (**B**) Open-field locomotion. Total distance traveled was not significantly altered in PERK cKO mice following systemic AAV-PHP.eB-Gfap-TSP1 administration (n = 6 mice/group; two-way ANOVA, effect of treatment: $F_{1, 100}$ = 3.094, P = 0.0817). (**C**) Mechanosensation: von Frey paw-withdrawal thresholds (PWT) were unchanged by astrocytic TSP1 expression (n = 6 mice/group; t_{10} = 0.4152, P = 0.6867; two-tailed unpaired t test). Summary data are mean ± SEM. ns, not significant; FC, fold change.

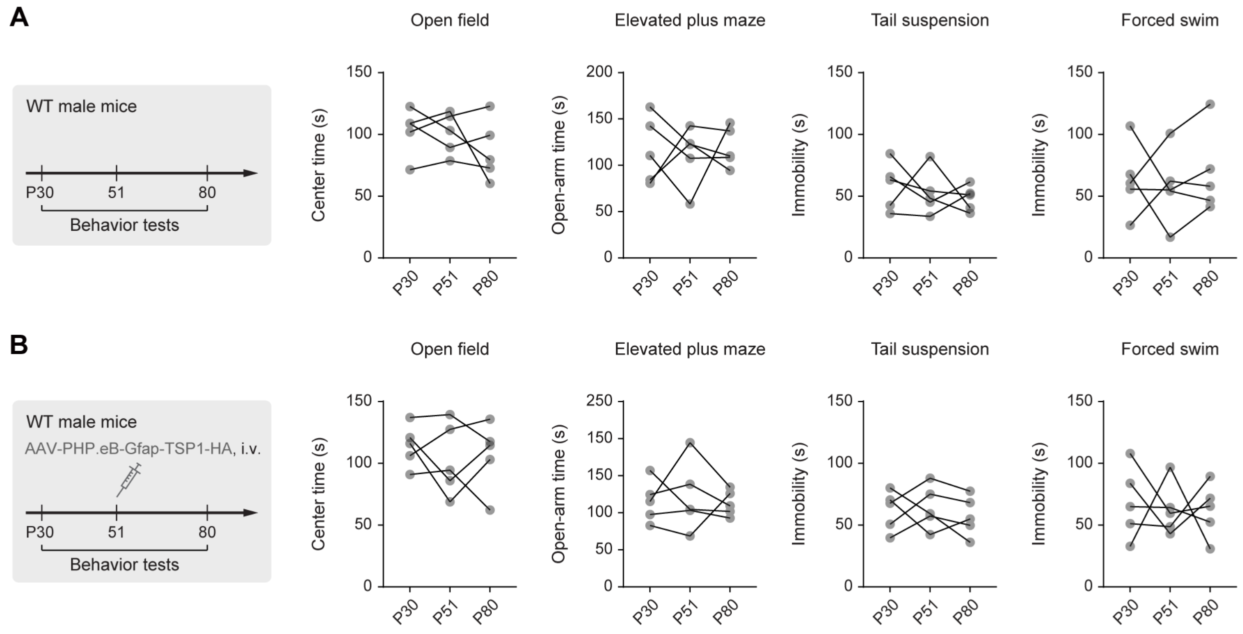

Figure S7. AAV-PHP.eB-mediated astrocytic TSP1 expression does not affect baseline behavior in unstressed WT mice

(**A**) Left, experimental timeline depicting three nonconsecutive behavioral sessions in WT male mice (n = 5 mice). Right, no significant effect of session was detected in the open field ($F_{2, 8}$ = 1.162, P = 0.3606), elevated plus maze ($F_{2, 8}$ = 0.07599, P = 0.9275), tail suspension test ($F_{2, 8}$ = 0.3889, P = 0.6900), or forced swim test ($F_{2, 8}$ = 0.1845, P = 0.8350). (**B**) Left, experimental timeline for a single systemic dose of AAV-PHP.eB-Gfap-TSP1 in WT male mice (n = 5 mice). Right, no significant effect of session was observed in the open field ($F_{2, 8}$ = 0.4294, P = 0.6650), elevated plus maze ($F_{2, 8}$ = 0.1165, P = 0.8915), tail suspension test ($F_{2, 8}$ = 0.265, P = 0.7737), or forced swim test ($F_{2, 8}$ = 0.07723, P = 0.9264). Each dot represents one mouse. RM one-way ANOVA.

Table S1. Human brain samples used for RNAscope analyses

Group	ID	Age	Brain Region	Manner of Death	Sex	Hemisphere	Clinical Brain Diagnosis
MDD	1	82	BA10	Natural	Female	Left	Major depressive disorder, recurrent, unspecified
MDD	2	76	BA10	Accidental	Female	Left	Major depressive disorder, recurrent, unspecified
MDD	3	85	BA10	Suicide	Female	Left	Major depressive disorder, recurrent, unspecified
MDD	4	57	BA10	Suicide	Female	Left	Major depressive disorder, recurrent, unspecified
MDD	5	41	BA10	Natural	Male	Left	Major depressive disorder, single episode, unspecified
MDD	6	83	BA10	Natural	Male	Left	Major depressive disorder, recurrent, unspecified
MDD	7	80	BA10	Natural	Male	Left	Major depressive disorder, recurrent, unspecified
MDD	8	56	BA10	Natural	Male	Left	Major depressive disorder, recurrent, unspecified
Control	1	67	BA10	Natural	Male	Left	No clinical brain diagnosis found
Control	2	54	BA10	Natural	Female	Right	No clinical brain diagnosis found
Control	3	66	BA10	Natural	Male	Left	No clinical brain diagnosis found
Control	4	95	BA10	Natural	Male	Right	No clinical brain diagnosis found
Control	5	88	BA10	Natural	Female	Left	No clinical brain diagnosis found
Control	6	61	BA10	Natural	Female	Right	No clinical brain diagnosis found
Control	7	84	BA10	Natural	Female	Left	No clinical brain diagnosis found
Control	8	65	BA10	Natural	Male	Right	No clinical brain diagnosis found

Table S2. Primary antibodies used in this study

Antibodies	Vendor	Catalog	Application (dilution)
PERK (D11A8)	Cell Signaling Technology	5683	WB (1:1000)
eIF2α	Cell Signaling Technology	9722	WB (1:1000)
Nrf2	Cell Signaling Technology	12721	WB (1:1000)
β-actin	Cell Signaling Technology	4967	WB (1:1000)
β-tubulin	Cell Signaling Technology	2146	WB (1:1000)
TUJ1	Cell Signaling Technology	5568	WB (1:1000)
GFAP	Abcam	ab4674	IF (1:1000)
GFAP	Invitrogen	130300	IF (1:1000)
S100β	Synaptic System	287 004	IF (1:1000)
S100β	Abcam	ab41548	IF (1:1000)
TSP1 (A6.1)	Thermo Fisher	MA513395	IF (1:200)
HA	Sigma	11867423001	IF (1:500)
IBA1	Synaptic System	234 308	IF (1:1000)
NeuN	Abcam	ab104224	IF (1:500)
NeuroTrace	Invitrogen	N21482	IF (1:500)
CD31	Abcam	ab28364	IF (1:500)
ZO-1	Proteintech	217731AP	IF (1:500)
PSD95 (6G6-1C9)	Sigma	MAB1596	IF (1:200)
Homer1	Invitrogen	PA521487	IF (1:200)
Puromycin	Sigma	MABE343	IF (1:500)

WB, Western blot; IF, immunofluorescence.

Table S3. RNAscope probes used in this study

Gene	Species	Vendor	Catalog #
GFAP	Human	ACD	311801-C2
EIF2AK3	Human	ACD	541471
SNAP25	Human	ACD	518851-C3
AIF1	Human	ACD	433121-C3
THBS1	Human	ACD	426581
Eif2ak3	Mouse	ACD	1041101-C1
S100β	Mouse	ACD	431731-C2
Thbs1	Mouse	ACD	457891
Aldh1l1	Mouse	ACD	405891-C2

Table S4. Primer sequences used for RT-qPCR

Thbs1	Forward	GGGGAGATAACGGTGTGTTTG
	Reverse	CGGGGATCAGGTTGGCATT
Gpc4	Forward	CTCAAGTCGAAAAGTTGCTCGG
	Reverse	CTTCAAATGGTCACCGTTGATCT
Gpc6	Forward	CCAATCAGGCGGATTTGGACA
	Reverse	GGGCCGAAAACGGGTGTTA
Thbs2	Forward	CTGGGCATAGGGCCAAGAG
	Reverse	GCTTGACAATCCTGTTGAGATCA
Sparcl1	Forward	GGCAATCCCGACAAGTACAAG
	Reverse	TGGTTTTCTATGTCTGCTGTAGC
Sparc	Forward	GTGGAAATGGGAGAATTTGAGGA
	Reverse	CTCACACACCTTGCCATGTTT
Chrdl1	Forward	AACCTCCAAGCCAAAACTTTGA
	Reverse	CCAGTGCTACTTTTCTGGTTGTC
Gapdh	Forward	AGGTCGGTGTGAACGGATTTG
	Reverse	TGTAGACCATGTAGTTGAGGTCA

Video S1. In vivo Ca^{2+} dynamics in mPFC pyramidal neurons and apical dendrites (Thy1-GCaMP6s). Three-minute continuous recording acquired via two-photon imaging in awake Thy1-GCaMP6s mice, targeting layer 5 pyramidal neuron somata and apical dendrites in the mPFC. Raw fluorescence frames are pseudo-colored using the “Magenta Hot” lookup table in ImageJ.
